# Dysregulated microRNAs in blood correlate with central nervous system neuropathology of prion disease

**DOI:** 10.1186/s13567-025-01566-0

**Published:** 2025-07-01

**Authors:** Sonia Pérez-Lázaro, Inmaculada Martín-Burriel, Luca Cozzuto, Julia Ponomarenko, Juan J. Badiola, Rosa Bolea, Janne M. Toivonen

**Affiliations:** 1 Centro de Encefalopatías y Enfermedades Transmisibles Emergentes (CEETE), Facultad de Veterinaria, Universidad de Zaragoza, Zaragoza, Spain; 2 Instituto Universitario de Investigación Mixto Agroalimentario de Aragón (IA2) UNIZAR-CITA, Zaragoza, Spain; 3 Instituto de Investigación Sanitaria de Aragón (IIS-Aragón), Zaragoza, Spain; 4 Laboratorio de Genética Bioquímica (LAGENBIO), Facultad de Veterinaria, Universidad de Zaragoza, Zaragoza, Spain; 5 Centro de Investigación Biomédica en Red de Enfermedades Neurodegenerativas (CIBERNED), Instituto de Salud Carlos III, Madrid, Spain; 6 Centro de Regulación Genómica (CRG), The Barcelona Institute of Science and Technology (BIST), Barcelona, Spain; 7 Universitat Pompeu Fabra (UPF), Barcelona, Spain

**Keywords:** Prion, neurodegenerative diseases, scrapie, microRNA, biomarkers

## Abstract

The role of microRNAs (miRNAs) in neurodegenerative diseases has gained significant attention because of their involvement in gene regulation and potential as biomarkers. In prion diseases, including scrapie, miRNAs may modulate pathogenesis and disease progression. This study investigated circulating miRNA profiles in the blood of sheep naturally affected by scrapie at preclinical and clinical stages using small RNA sequencing and RT-qPCR validation. While only one novel miRNA was dysregulated in preclinical blood samples, 66 previously annotated miRNAs were significantly dysregulated in clinical sheep compared with healthy sheep. These miRNAs are associated with pathways commonly altered in neurodegenerative diseases, such as autophagy, ubiquitin-mediated proteolysis, and endoplasmic reticulum protein processing. Notably, miR-1271-5p, let-7f-5p, miR-186-5p, and miR-425-5p were consistently upregulated in the central nervous system of clinical animals, replicating the results observed in blood, with an increasing trend already in the preclinical stage and a strong correlation with neuropathological prion features. Additionally, predicted target genes such as *UBQLN2*, *PGK1*, *KRAS*, and *CLTC* were inversely expressed relative to these miRNAs, supporting their regulatory roles. These findings highlight the relevance of circulating miRNAs in prion neuropathology and support further research into the specific functional roles of these miRNAs and their predictive capacity for disease progression.

**Supplementary Information:**

The online version contains supplementary material available at 10.1186/s13567-025-01566-0.

## Introduction

Prion diseases, also known as transmissible spongiform encephalopathies (TSEs), represent a group of complex neurodegenerative conditions that affect both animals and humans. These disorders are caused by a misfolded protein (PrP^Sc^), which acts as an infectious agent by inducing the abnormal folding of the physiological cellular prion protein (PrP^C^). PrP^C^, encoded by the *PRNP* gene, is highly expressed in the brain and plays a role in protection against oxidative stress, synaptic transmission and neuronal homeostasis [1]. However, its conversion into PrP^Sc^ causes the formation of non-degradable aggregates and their accumulation, primarily in the central nervous system (CNS). The deposition of these aggregates leads to progressive neurodegeneration and neuronal damage, ultimately resulting in the death of the individual. Common neuropathological findings in the CNS include PrP^Sc^ deposits, spongiform vacuolation and activation of astrocytes and microglia [2]. Animal prion diseases include scrapie in sheep and goats, the first TSE described, bovine spongiform encephalopathy in cattle and chronic wasting disease (CWD) in cervids. In humans, Creutzfeldt‒Jakob disease is the most prevalent disease, with its sporadic nature (sCJD) accounting for most cases [3]. In addition to prion diseases, a group of neurodegenerative pathologies commonly called prion-like disorders are characterised by the accumulation of protein aggregates, such as beta-amyloid and tau in Alzheimer’s disease (AD), alpha-synuclein in Parkinson’s disease (PD) and SOD1 and TDP-43 in amyotrophic lateral sclerosis (ALS) [4, 5].

While the pathological hallmarks of prion diseases are well documented, the molecular mechanisms underlying the onset and progression of these diseases, especially during the asymptomatic preclinical stage, remain poorly understood. Early detection before the appearance of clinical signs is essential for understanding disease progression.

Currently, the detection of PrP^Sc^ in post-mortem brain tissue is the only definitive method for the diagnosis of prion diseases. In classical scrapie, however, preclinical diagnosis is possible in vivo through rectal mucosa biopsies because of the spread of PrP^Sc^ throughout the lymphoid tissue at the beginning of the pathogenesis of the disease [6]. Although this technique is not 100% sensitive for diagnostic use, as the involvement of the lymphoid system depends on many factors, it is fully specific and provides us with an animal model of natural prion disease in which to investigate all the molecular changes occurring in the preclinical stage. In this context, sheep scrapie has previously been used as a faithful model for human prion diseases [7, 8].

In recent years, microRNAs (miRNAs) have emerged as key regulators of numerous diseases. These small (20–25 base pairs long), noncoding nucleotides can regulate gene expression post-transcriptionally, binding to complementary messenger RNA (mRNA) sequences through the RNA-induced silencing complex (RISC) and thus degrading, destabilising or translationally inhibiting target mRNAs [9]. It is predicted that approximately 70% of the identified miRNAs are expressed in the brain and that they may regulate up to 30% of protein-coding genes [10, 11]. The expression and regulatory potential of these genes suggest that they may play important roles in the pathogenesis of neurodegenerative disorders, including prion diseases.

Particularly in prion diseases, previous research has identified several miRNAs that may directly regulate *PRNP* expression, and moreover, the prion protein has also been predicted to interact with miRNA biogenesis pathways [12]. Possibly related to this direct regulation, Norsworthy and colleagues identified a miRNA signature capable of differentiating sCJD from AD, although these miRNAs did not correlate with clinical parameters or disease progression [13]. These findings indicate that further research is needed to understand the functional role of these proteins in the neuropathogenesis of prion diseases. Studies using small RNA sequencing in the CNS of preclinical murine models of prion disease have revealed that only a limited number of miRNAs are altered in early stages, suggesting that miRNA dysregulation may occur in advanced stages of the disease [14, 15]. In naturally occurring scrapie, research has been limited to specific miRNAs and has focused only on the clinical stage of disease, leaving unanswered what happens in naturally occurring disease before the onset of clinical signs or whether the expression of miRNAs correlates with prion neuropathology [16, 17].

Interestingly, miRNAs exhibit remarkable stability in body fluids, such as blood and cerebrospinal fluid (CSF), due to their encapsulation in extracellular vesicles or their association with RNA-binding proteins, making them ideal fluid biomarkers [9, 18]. Blood is a highly accessible and minimally invasive body fluid, offering significant advantages for early diagnosis and disease monitoring. miRNAs detected in blood often originate from various tissues, potentially providing insights into otherwise inaccessible brain pathologies [19]. Nevertheless, only one study in a prion murine model has identified commonly dysregulated miRNAs between body fluids and the CNS, highlighting the need for further research to identify reliable biomarkers in peripheral fluids that could reflect what is happening in the brain during disease progression [20].

In this study, we aimed to investigate whether miRNA dysregulation in blood could serve as a biomarker for disease progression, with a specific focus on a natural model of prion disease. We also sought to test the hypothesis that miRNA dysregulation appears predominantly in the clinical stages, as suggested by prior studies in murine prion models. Given these objectives, we performed next-generation small RNA sequencing to profile, for the first time, miRNA changes in blood from healthy sheep through preclinical and clinical stages of naturally affected scrapie sheep. After validating significantly dysregulated miRNAs in the blood via real-time quantitative PCR (RT-qPCR), we also analysed these miRNAs in the CNS and correlated their expression with the characteristic neuropathology of prion diseases. Furthermore, we investigated the expression of potential mRNA targets of dysregulated miRNAs to gain insights into their role in neurodegeneration.

## Materials and methods

### Animals

A total of 25 female Rasa Aragonesa sheep (3–6 years old), which carry the ARQ/ARQ *PRNP* genotype, one of the most prevalent *PRNP* genotypes in scrapie-affected Rasa Aragonesa sheep [21], were included in this study. These sheep were categorised into three groups according to their scrapie-affection status and disease progression: 10 healthy sheep, 5 naturally scrapie-affected sheep in the preclinical stage displaying no clinical signs and 10 scrapie-affected sheep in the clinical stage of the disease. All sheep were bred under natural flock conditions, and it is expected that their reproductive status would be similar across groups. None of the animals were lactating at the time of sampling. Body condition scores were assessed using a standard 1–5 scale (1 = very thin; 5 = obese) by palpation of the lumbar region, with values ranging from 2.5 to 3.5 in healthy and preclinical animals. Healthy animals were used as the control group and were from healthy flocks with no previous scrapie cases. Animals naturally affected with scrapie were selected from flocks involved in scrapie outbreaks. Specifically, clinical sheep were identified by characteristic scrapie clinical signs such as pruritus coursing with alopecic areas around the lumbar region, cachexia -with body condition scores ranging from 1.5 to 2.25, consistent with disease progression- and ataxia [22], and confirmed by the detection of PrP^Sc^ deposits in CNS samples using immunohistochemistry (IHC), as detailed in the histopathological and immunohistochemical analyses section below. Furthermore, preclinical animals with no clinical signs were detected by large-scale rectal mucosa biopsies and subsequent IHC, as described elsewhere [23].

### Blood and brain tissue sample collection

Blood samples (2.5 mL) were collected by jugular vein puncture into PAXgene Blood RNA Tubes (PreAnalytiX, Switzerland) to better preserve intracellular RNA for molecular procedures. The tubes were filled, inverted 10 times and, after two hours at room temperature (RT), frozen at −80 °C until processing. The animals were subsequently sacrificed via intravenous injection of sodium pentobarbital, followed by exsanguination. Samples from two areas of the CNS (medulla oblongata at the level of the obex and thalamus) were divided in half sagittally; one half was finely minced and preserved in RNAlater Solution (Invitrogen, USA) at −80 °C for posterior RNA extraction, while the other half was fixed in 10% formaldehyde for histopathological and immunohistochemical analyses.

### Histopathological and immunohistochemical analyses

Formalin-fixed obex and thalamus tissues were paraffin-embedded, sliced into 4 µm thick sections and dried at 37 °C for 24 h. Haematoxylin and eosin (HE) staining was performed to assess neuropathological changes and spongiosis degeneration. PrP^Sc^ deposits in the CNS were quantified by IHC with an L42 antibody (1/500, R8005, R-Biopharm, Germany) following an established protocol [24]. Astrogliosis and microgliosis were also evaluated by IHC, using glial fibrillary acidic protein (GFAP; 1/500, Z0334, Dako, Denmark) as an astrocyte marker and ionised calcium-binding adaptor molecule 1 (Iba1; 1/1000, 019-19741, Wako, USA) as a microglial marker. Previously reported protocols were followed for both [25]. Blinded histopathological and immunohistochemical evaluations were performed using a Zeiss Axioskop 40 optical microscope (Zeiss, Germany). Microphotographs of representative areas of each region (thalamus: dorsal, central, ventral; medulla oblongata at the level of the obex: cuneate nucleus, dorsal motor nucleus of vagus, inferior olive) were taken using an Axiocam 305 color camera (Zeiss, Germany) and Zeiss Zen v3.9 software, ensuring the same intensity and contrast settings across all samples. The staining intensity was analysed via ImageJ v1.54 g software with the Colour Deconvolution 2 v2.1 plugin [26], following a previously described method [27]. Briefly, colour deconvolution vectors appropriate for each staining type (H DAB for IHC or H&E for HE) were selected accordingly, and a specific threshold was set manually to remove background signals and uniformly applied to each neuropathological feature for consistency across samples. The mean grey values were then measured and averaged by area and sample for statistical analysis and graphical representation. In the IHC samples, higher mean grey values indicated greater staining intensity, corresponding to higher expression of the proteins marked, whereas in the HE-stained samples, a higher mean grey value reflected greater spongiosis.

### Total RNA extraction

Total RNA was extracted from the entire volume of whole blood collected using the PAXgene Blood RNA Kit (PreAnalytiX, Switzerland) following the manufacturer’s steps. Eighty microliters of RNA was obtained and stored at -80 °C until use. A Direct-zol RNA Miniprep Plus Kit (Zymo Research, USA) was used for RNA extraction from obex and thalamus preserved tissues. Twenty milligrams of tissue was homogenised in 1 mL of TRI Reagent (Zymo Research, USA) using TeSeE grinding tubes and a TeSeEPrecess 48 homogeniser (Bio-Rad, USA). After the samples were left for 5 min at 4 °C and for another 5 min at RT, chloroform phase separation was performed by adding 200 µL of chloroform, shaking for 15 s, incubating for 3 min at RT and centrifuging at 12 000 *g* at 4 °C for 15 min. After this, the colourless upper aqueous phase was mixed with an equal volume of ethanol and transferred into the kit columns. The manufacturer’s protocol was followed from this point onwards for RNA purification. The RNA was eluted with 50 µL of nuclease-free water and stored at −80 °C until analysis.

### Small RNA sequencing

RNA quality control and concentration measurements were performed using a NanoDrop spectrophotometer (Thermo Fisher Scientific, USA) and a 2100 Bioanalyzer (Agilent, USA). All the RNA integrity number (RIN) values were above 8. Blood RNA samples were sent to the Genomics Unit at the Centro de Regulación Genómica (CRG, Barcelona, Spain), where small RNA sequencing was performed. The sequencing libraries were prepared using the NEBNext Multiplex Small RNA Library Prep Set for the Illumina Kit (New England Biolabs, USA) following the manufacturer’s protocol. Fifty bp (base pairs) single-end reads were sequenced on the Illumina HiSeq 2500 platform (Illumina, USA).

### Bioinformatic data analysis

The raw reads were quality-checked using FastQC v0.11.7 [28] to assess the read quality, adapter contamination and GC content. Sequencing adapters and reads for quality and length were trimmed using Skewer v0.2.2 [29], with default parameters. Reads between 15 and 30 bases in length were aligned to the sheep reference genome (Oar_v4.0) using ShortStack v3.8.5 [30], which is based on Bowtie 1 v1.2.2 [31]. MiRNAs were annotated using *Ovis aries* miRNAs in miRBase v22 [32], and reads were quantified using HTSeq-count [33]. Owing to the limited number of *Ovis aries* miRNAs in miRBase, a high percentage of reads were not assigned. These reads were subjected to de novo prediction using Infernal v1.1.4 [34] with the whole Rfam database v14.6 [35], preselecting miRNAs with at least ten reads across all samples. Additionally, miRDeep2 v2.0.1.2 [36] was employed to detect mature miRNAs from other species available in miRBase, and to predict novel miRNAs. The secondary structure of novel miRNAs was predicted using RNAfold from the ViennaRNA package v2.0 [37], and the minimum folding energy (MFE) of the structure was evaluated against a distribution obtained by shuffling input sequences while preserving dinucleotide composition, using RANDfold [38].

Infernal and miRDeep2 predictions were combined with the annotated miRNAs in *Ovis* arrays from miRBase and filtered to remove redundancy using Bedtools v2.27.1 [39] to generate a final annotation. Merged annotation reads were quantified using HTSeq-count and filtered to retain miRNAs with at least one read per sample per group. Specifically, miRNAs were retained if they had at least ten reads in the clinical and healthy groups each and five reads in the preclinical group. Differential expression analysis was performed using DESeq2 v1.34 [40]. Sequences from differentially expressed novel miRNAs were queried against the RumimiR database [41] using the integrated BLAST tool to evaluate whether any of those miRNAs had been previously reported in other ruminant studies. We considered only those hits with an E value lower than 0.001 to be highly accurate, and priority was given to sheep studies, all of which used the same reference sheep genome, Oar_v4.0, when available.

Unsupervised multivariate analysis was conducted using principal component analysis (PCA) in R v4.2.2, with the ggfortify v0.4.16 package, to assess the small RNA sequencing data distribution across the three experimental groups on the basis of logarithmically transformed read counts. A Venn diagram was created using the VennDiagram v1.7.3 R package, and volcano plots were designed with the ggplot2 v3.4.2 R package. Additionally, hierarchical clustering analysis was performed using the ComplexHeatmap v2.14.0 R package. Fold change (FC) values were calculated from selected miRNA logarithmically transformed read counts, referring each scrapie-affected stage to the mean of the healthy group. Z scores were calculated by scaling FC values, and hierarchical clustering was conducted using Euclidean distance and a complete linkage method. Functional enrichment analysis based on the Kyoto Encyclopedia of Genes and Genomes (KEGG) pathways was performed using DIANA-miRPath v4.0 [42], which uses human databases for target prediction with TarBase v8.0, and the union merging method for genes was selected.

### miRNA RT-qPCR analyses

For both blood and CNS samples, complementary DNA (cDNA) was synthesised using the TaqMan MicroRNA Reverse Transcription Kit (Thermo Fisher Scientific, USA) following the manufacturer’s instructions. Thirty nanograms of RNA was used as a template, and two reverse transcription reactions were performed, pooling separately TaqMan MicroRNA RT Assays (Thermo Fisher Scientific, USA) for the miRNAs of interest and for potential housekeeping miRNAs and adding two negative controls with no reverse transcriptase or no template to test the reaction.

RT-qPCR was performed on a QuantStudio 3 instrument (Thermo Fisher Scientific, USA). Reactions were run in triplicate and consisted of 5 µL of TaqMan Fast Universal Master Mix (2X) without AmpErase UNG (Thermo Fisher Scientific, USA), 4.5 µL of 1/5-diluted cDNA and 0.5 µL of TaqMan MicroRNA TM Assays (Thermo Fisher Scientific, USA) for each miRNA analysed. Housekeeping miRNA candidates were selected from the blood small RNA sequencing data. Additionally, sn-U6 was analysed as a potential housekeeping gene only in the CNS because of its previously reported stability in this type of sample [14]. The TaqMan probe references are detailed in Additional file 1. Cycle threshold (Ct) values were calculated using the QuantStudio Design & Analysis software v1.5.3 (Thermo Fisher Scientific, USA), and Ct values below 35 were considered acceptable. Blood and obex samples were normalised to the mean of miR-92a and miR-328, whereas thalamic samples were normalised to the mean of sn-U6, miR-320 and miR-328, identified as the most stable miRNAs among groups by RefFinder [43], with no significant Ct differences across groups (Additional file 2). For RT-qPCR results analysis, the 2^−∆∆Ct^ method was used, with the healthy control group used as the reference group [44]. Briefly, delta Ct values were calculated by subtracting the mean housekeeping Ct values from the selected miRNA Ct values for each sample. The mean of the delta Ct values of all healthy controls was subsequently subtracted from each sample to obtain the delta Ct values, and the calculated 2^−∆∆Ct^ values were graphed as individual dots.

### miRNA target identification and expression analyses

Since there are no sheep-specific miRNA target databases, human databases were used after confirming miRNA sequence conservation. Target genes for selected miRNAs likely to be involved in prion pathology were evaluated using TarBase v9.0 [45], considering high-confidence miRNAs with primary interactions only, and filtering for genes validated by a luciferase assay and those validated by immunoprecipitation in more than 5 experiments; miRDB v6.0 [46], filtering results with a target prediction score higher than 80; and DIANA-miRPath v4.0 [42], preselecting target genes related to neurodegenerative pathways. Then, intersections between these databases were performed to refine the target genes. Additionally, two previous articles that used microarrays and mass spectrometry analyses for identifying biomarkers in sheep naturally affected with scrapie were also considered [47, 48] and filtered for genes or proteins inversely expressed to the miRNAs selected. We confirmed the conservation of miRNA‒target gene binding sites in sheep, using the alignment comparative genomics tool in Ensembl [49].

For gene expression quantification by RT-qPCR, 1 µg of total RNA was reverse transcribed into cDNA using the qScript cDNA SuperMix Kit (Quantabio, USA) according to the manufacturer’s instructions. Primers were designed using Primer3Plus v3.3.0 [50] and PrimerBLAST [51] tools, ensuring separation by an intron when possible, total specificity for the gene in *Ovis aries* and minimal secondary structures between primers. The primers were then tested for their ideal concentration and efficiency, with 90–110% efficiency. *GAPDH*, *G6PD* and *SDHA* were analysed as potential housekeeping genes for their previously reported stability in the CNS of scrapie-affected sheep [52]. The primer sequences and concentrations used are detailed in Additional file 3.

RT-qPCR was performed in triplicate on a QuantStudio 3 instrument (Thermo Fisher Scientific, USA) with the following reaction mixture: 5 µL of RealQ Plus 2 × Master Mix Green, low ROX (Ampliqon, Denmark), variable primer concentration (as shown in Additional file 3) and 15 ng of cDNA. Standard run mode was used, with a melt curve used to check for specific amplification. The target gene data in both the obex and thalamus areas were normalised to the means of *G6PD* and *SDHA* because of their better stability, as reported by RefFinder, and their lack of significant differences in Ct values between groups (Additional file 4). The delta Ct values were calculated, and the 2^−∆∆Ct^ method was subsequently used as described for the miRNAs.

### Statistical analyses

All the statistical analyses and graphical representations were performed in R version 4.2.2.

Small RNA sequencing *p* values were adjusted (adj *p* value) via Benjamini‒Hochberg false discovery rate (FDR) correction for multiple testing. For statistical comparisons between groups, we first used the Shapiro‒Wilk test to check data normality. If the data were normal, and after testing for homogeneity of variance with Levene’s test, the variables were compared using one-way ANOVA, followed by the Tukey HSD test for multiple pairwise comparisons. Non-normally distributed or non-homogeneous variables were tested using the Kruskal‒Wallis test, followed by Dunn’s test for multiple pairwise comparisons. Normally distributed variables are described as the mean ± standard deviation, and non-normally distributed variables are described as the median ± interquartile range. For the correlation analysis between miRNA expression levels in the CNS and neuropathological changes, the cor R function and the Hmisc v5.1.1 R package were used for the calculation of Spearman’s rank correlation coefficients and correlation *p* values, respectively. This analysis was performed separately for each CNS area, using the 2^−∆∆Ct^ values for miRNA expression and the ImageJ mean grey value scores for neuropathology.

Data visualisation was conducted using the ggplot2 package in R, and statistically significant differences are indicated in the plots by symbols, where tendencies are marked with ^ (*p* value < 0.1) and significant results with asterisks: * (*p* value < 0.05), ** (*p* value < 0.01) and *** (*p* value < 0.001).

## Results

### Neuropathological features of naturally occurring scrapie stages

**Figure 1:**
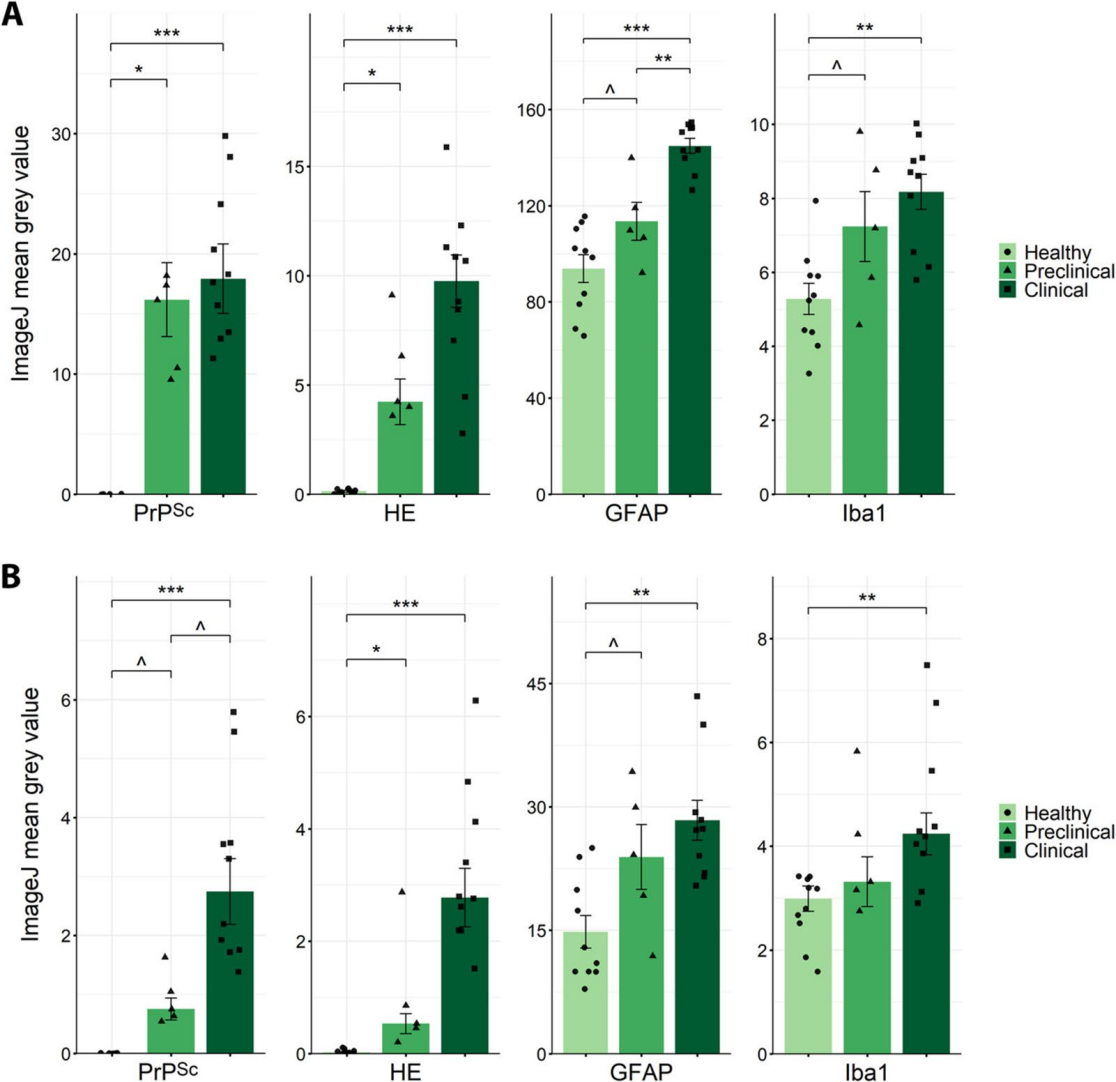
**Neuropathological features in the obex (A) and thalamus (B).** Scatter plots representing the mean grey values calculated by ImageJ, using representative microphotographs of each area and a consistent threshold for all samples in each neuropathological feature analysed: PrP^Sc^ deposits (PrP^Sc^), spongiosis (HE), astrogliosis measured with glial fibrillary acidic protein (GFAP), and microgliosis measured using ionised calcium-binding adaptor molecule 1 (Iba1). The bars and error lines indicate the means of individual values and standard deviations or the medians and interquartile ranges for normally and non-normally distributed data, respectively. For each neuropathological feature, significant differences between healthy (light green and circles, *n* = 10) and preclinical (green and triangles, *n* = 5) and clinical (dark green and squares, *n* = 10) naturally affected scrapie groups were measured using one-way ANOVA followed by Tukey’s HSD (normally distributed data) or the Kruskal‒Wallis test followed by Dunn’s test (non-normally distributed). *p value < 0.05, **p value < 0.01, ***p value < 0.001, ^*p* value < 0.1 (tendency).

First, we analysed the characteristic neuropathological features of prion diseases in all the samples included in this study: 5 preclinical naturally scrapie-affected sheep, 10 clinical scrapie sheep and 10 healthy animals (Figure 1). PrP^Sc^ deposits, spongiosis (HE staining), astrogliosis (GFAP) and microgliosis (Iba1), were evaluated in two areas of the CNS previously reported to be the most affected by scrapie: the medulla oblongata at the level of the obex and the thalamus [53]. Similar pathological differences between groups were observed in both CNS regions, with the obex showing greater intensity of these neuropathological features appearing to be more affected. PrP^Sc^ deposits and spongiosis are absent in healthy animals, as these are hallmark features of prion diseases, and both are significantly elevated in scrapie-affected animals and are already altered in the preclinical stage of disease. GFAP and Iba1 reactivity was also significantly increased in clinical animals in both CNS areas studied. Additionally, these markers tended to be more highly expressed in preclinical sheep than in healthy animals, particularly in the obex, whereas in the thalamus, only GFAP displayed early upregulation.

### Small RNA sequencing reveals notable miRNA dysregulation in scrapie blood

**Figure 2:**
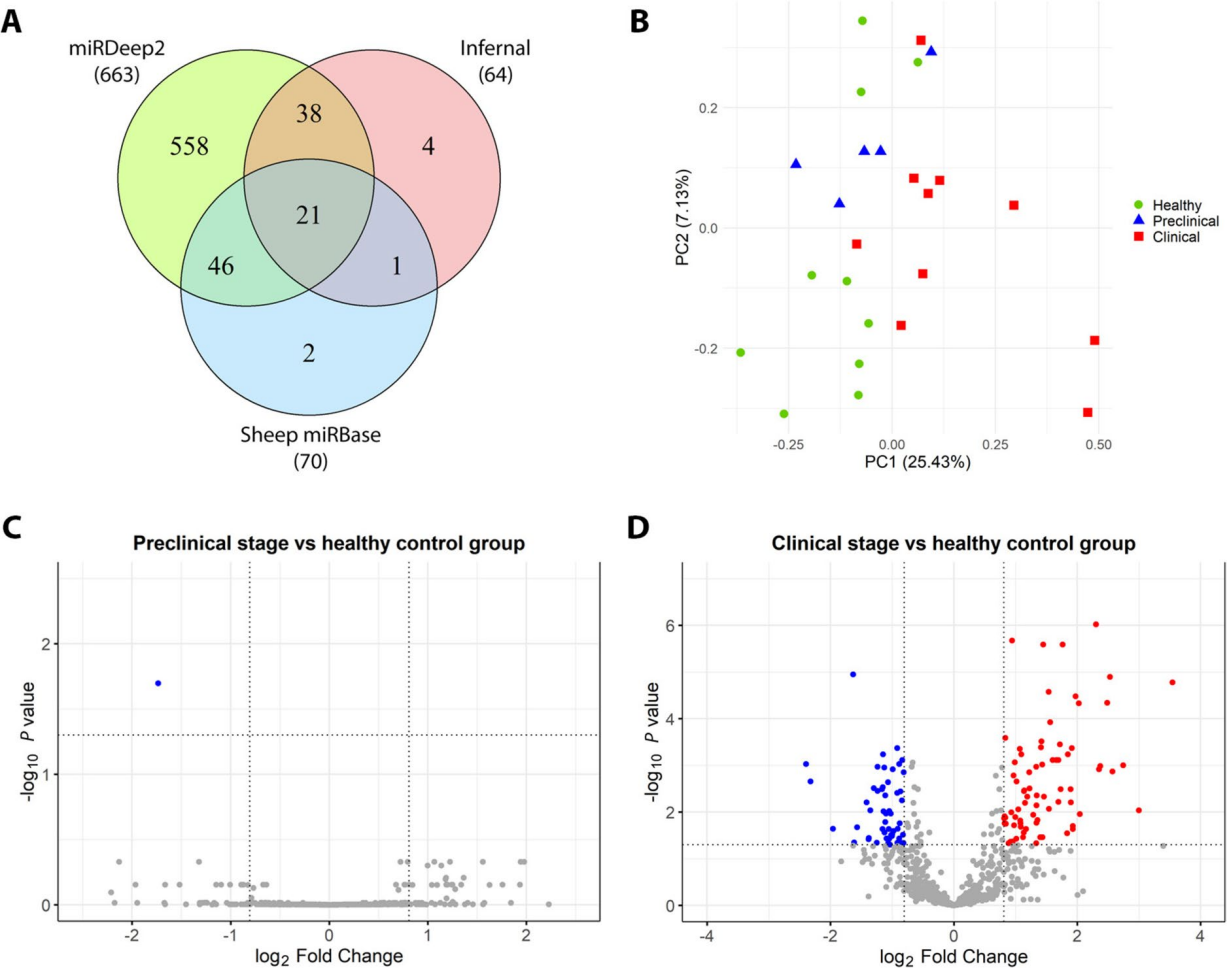
**Data exploration of the small RNA sequencing results**. **A** Venn diagram representing miRNAs identified by miRDeep2, Infernal and sheep miRBase and their overlap after filtering the small RNA sequencing results for minimum expression levels. **B** Principal component analysis (PCA) of normalised small RNA sequencing read counts from all blood samples. The x- and y-axes represent two principal components (PC1 and PC2), which account for 25.43% and 7.13% of the variance between samples, respectively. The data points represent healthy animals (green circles, *n* = 10) and preclinical (blue triangles, *n* = 5) and clinical (red squares, n = 10) scrapie-affected sheep. **C and D**, Volcano plots showing differential blood miRNA expression in preclinical (**C**) and clinical stages (**D**) compared with healthy controls. The horizontal line shows an adjusted *p* value of 0.05, whereas the vertical lines show log_2_-fold change values of -0.8 and + 0.8. Significantly downregulated miRNAs are coloured in blue, significantly upregulated miRNAs are coloured in red, and non-differentially expressed miRNAs are coloured in grey.

Next-generation sequencing was performed on blood samples from naturally affected scrapie animals and healthy controls to measure miRNA expression and detect potential differences between diseased and healthy animals. All the samples yielded an average of 6.9 million quality-controlled reads (ranging from 4.4 to 9.4 million reads per sample). The initial analysis aligned almost 96% of the total reads to the sheep reference genome and focused on known miRNAs annotated in the miRBase for sheep. However, the percentage of reads assigned to known miRNAs from sheep was notably low, with a mean assignment rate of less than 3%. Consequently, we used de novo prediction of miRNAs, assigning, on average, almost 90% of the reads to the predicted miRNAs across samples. As a result, 1097 putative miRNAs were identified. After filtering for minimum expression, a total of 670 miRNAs remained. Figure 2A shows the distribution of these miRNAs identified by three bioinformatic tools. Approximately 80% of the miRNAs were identified uniquely by miRDeep2; among these, 106 were detected by miRDeep2 as mature miRBase miRNAs from other species, whereas 452 were predicted as novel miRNAs.

Using these filtered miRNAs, PCA was performed to graphically represent the overall differences in miRNA expression between samples (Figure 2B). We subsequently performed differential expression analyses between the groups. After FDR correction for multiple testing, only one putative novel miRNA was found to be significantly downregulated (adjusted *p* value < 0.05) in the preclinical stage group compared with the healthy control group (Figure 2C, Additional file 5). This miRNA was predicted by miRDeep2 and has not been previously described in other species. Its precursor sequence was mapped to chromosome 7 in the *Ovis aries* genome and exhibited a characteristic stem‒loop structure. The predicted MFE probability of 0.045 suggested a low likelihood of random folding, supporting its classification as a true miRNA precursor (Additional file 6).

In the comparison of the clinical stages of the scrapie and healthy control groups, 126 miRNAs were found to have an adjusted *p* value < 0.05 and an absolute value of log_2_ FC > 0.8 (Figure 2D). Among these, 60 miRNAs were predicted to be novel miRNAs by miRDeep2 (Additional file 7). The remaining 66 known miRNAs included 63 that were significantly upregulated and 3 that were significantly downregulated in clinical sheep (Additional file 8). Among the 60 miRNAs predicted as novel, 28 showed high sequence similarity (E value < 0.001) to entries in the RumimiR database, suggesting that they had been previously detected in other ruminant studies (Additional file 9).

### Functional enrichment analysis highlights neurodegeneration-related pathways

To further investigate the biological processes in which significantly dysregulated miRNAs might be involved, we performed a KEGG pathway enrichment analysis using DIANA-miRPath. Among the total 66 known miRNAs significantly dysregulated in the clinical stage, only 57 miRNAs were mapped to the human miRPath database. We obtained 85 significantly enriched KEGG pathways (adjusted *p* value < 0.01) after filtering for pathways with at least 55 input miRNAs involved (Additional file 10). The top 20 most significantly enriched pathways are listed in Table 1. These include proteolysis, autophagy and multiple neurodegenerative conditions.

**Table 1:** **Top 20 KEGG pathways associated with significantly dysregulated** **(adjusted** ***p*** **value < 0.01) miRNAs in blood.**

KEGG pathway	adj *p* value^1^	Target genes (n)	miRNAs (n)
Ubiquitin mediated proteolysis	3.64E-19	124	56
Pathways in cancer	9.90E-17	375	56
Shigellosis	1.61E-15	200	56
Autophagy—animal	1.24E-14	120	55
Cell cycle	6.24E-13	106	56
Proteoglycans in cancer	2.56E-12	163	57
FoxO signalling pathway	4.83E-12	111	56
Renal cell carcinoma	2.31E-11	63	55
Amyotrophic lateral sclerosis	1.03E-10	270	56
Protein processing in endoplasmic reticulum	1.11E-10	143	55
Salmonella infection	2.96E-10	192	56
Pathways of neurodegeneration—multiple diseases	6.36E-10	341	56
Chronic myeloid leukemia	1.21E-09	67	56
Focal adhesion	2.71E-09	151	56
HIF-1 signalling pathway	3.95E-09	88	55
Huntington disease	5.25E-09	224	56
Colorectal cancer	5.35E-09	72	56
p53 signalling pathway	7.47E-09	63	56
Neurotrophin signalling pathway	7.47E-09	95	55
Prostate cancer	9.96E-09	80	55

^1^adj *p* value: *p* value adjusted via Benjamini‒Hochberg false discovery rate correction

### Blood miRNA validation by RT-qPCR

**Figure 3:**
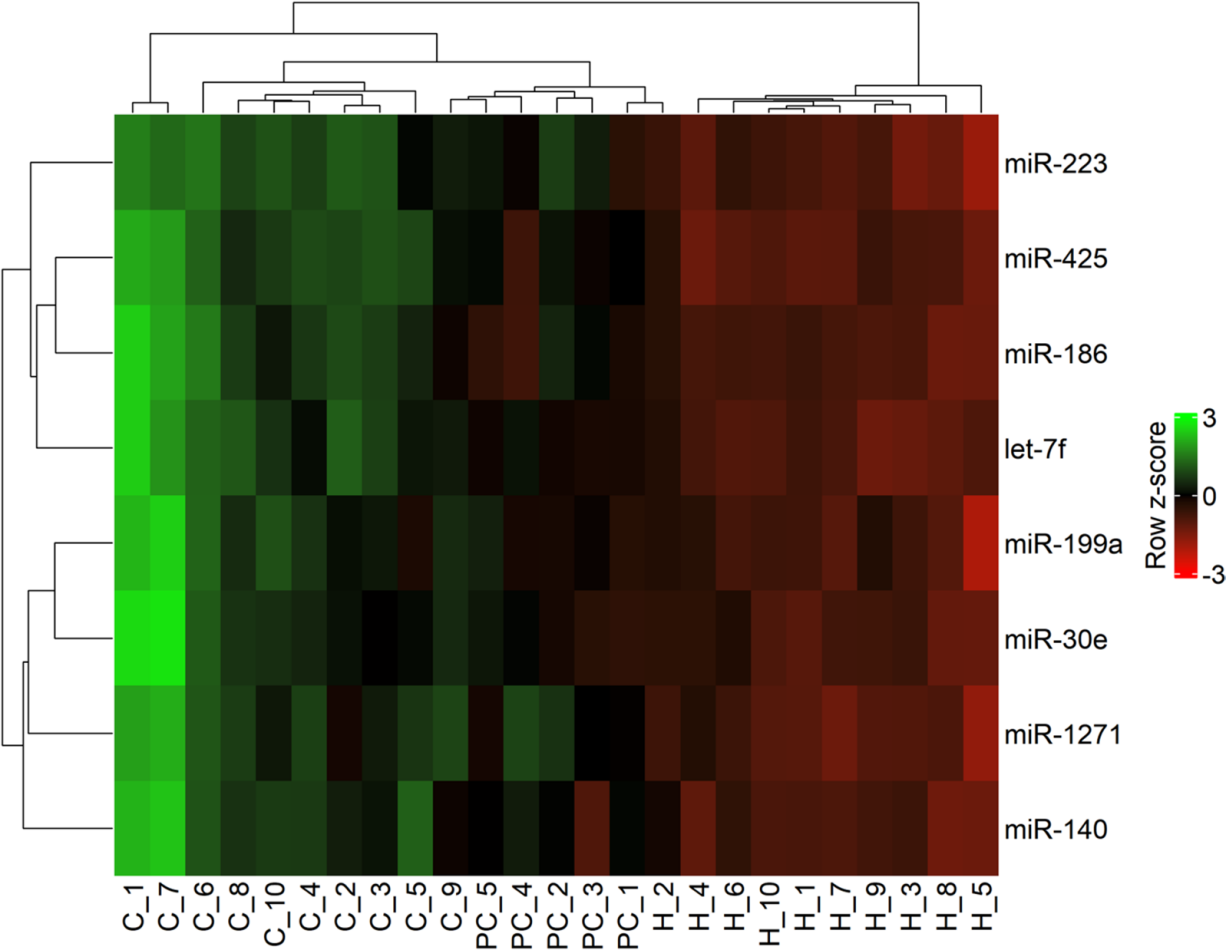
**Hierarchical clustering analysis of selected miRNAs in blood.** Relative differential expression of selected miRNAs (in rows), presented as z scores calculated from normalised small RNA sequencing read counts of all blood samples (in columns; C: clinical stage of naturally scrapie-affected sheep, PC: preclinical stage, H: healthy controls). Dendrograms in the y- and x-axes show clustering of miRNAs and samples, respectively, using a complete linkage method and the Euclidean method for distance measurement.

To select miRNAs for RT-qPCR validation, we prioritised those that were upregulated in the clinical stage group compared with the healthy control group, since the majority (63 out of 66) of the known significantly dysregulated miRNAs identified in this comparison were overexpressed in clinical animals. Our selection criteria included greater statistical significance, a fold change in differential expression, and a median read count above 100 in both groups studied to ensure reliable detection by RT-qPCR, given the sensitivity limitations of this validation technique. On the basis of these parameters and the exploratory nature of this study, eight miRNAs were selected for further validation by RT-qPCR. Figure 3 represents a hierarchical clustering analysis with the relative expression of each of the selected miRNAs from the small RNA sequencing data. The clusters in which the samples are grouped according to the expression of the selected miRNAs in each sample resemble the groups studied.

**Figure 4:**
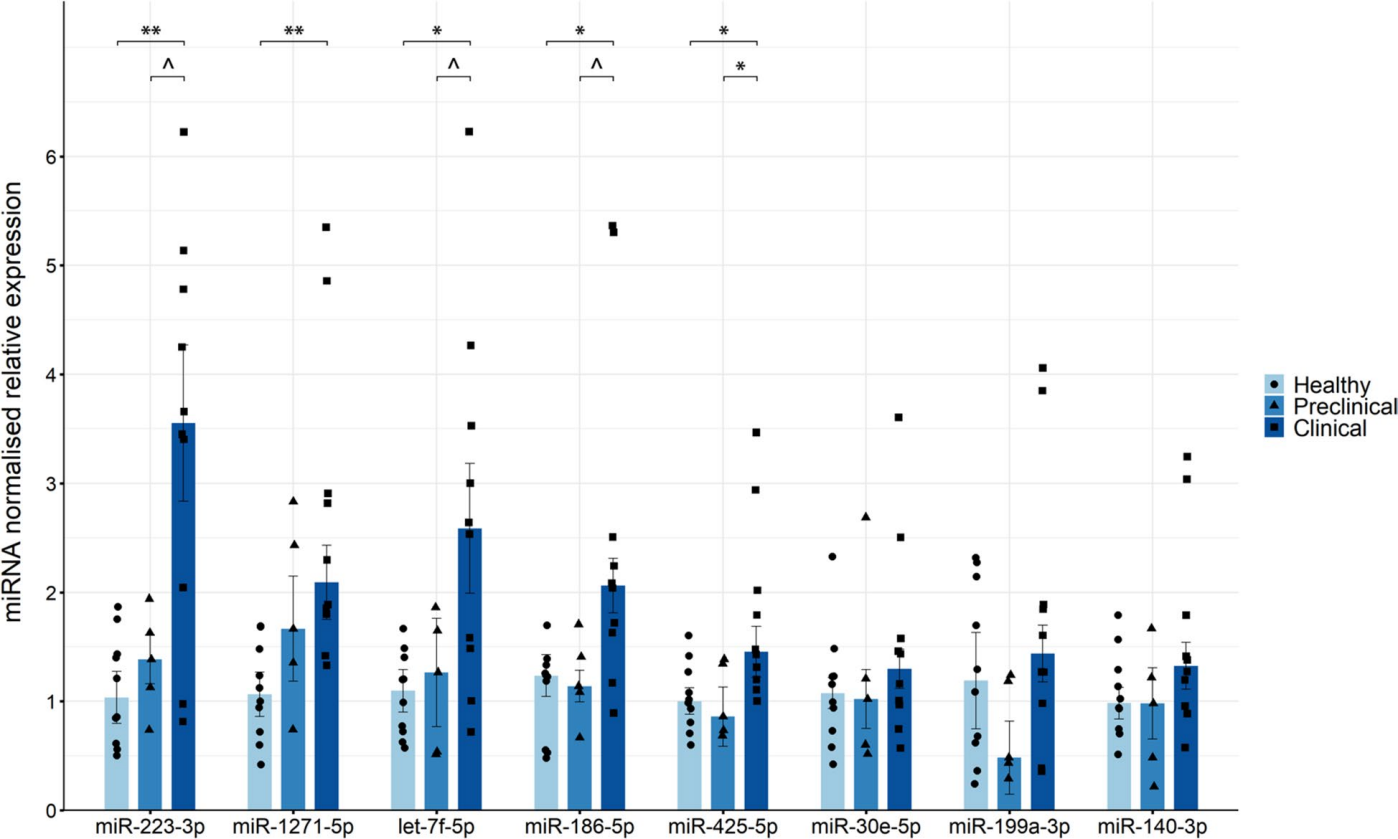
**Relative expression of selected miRNAs measured by RT-qPCR in blood.** Grouped bar plot with overlaid scatter plot showing individual 2^−∆∆Ct^ values relative to the healthy group. The bars and error lines indicate the means of individual values and standard deviations or the medians and interquartile ranges for normally and non-normally distributed data, respectively. miRNA expression was normalised to the mean expression of miR-92a and miR-328. For each miRNA, significant differences between healthy (light blue and circles, *n* = 10) and preclinical (blue and triangles, *n* = 5) and clinical (dark blue and squares, *n* = 10) naturally affected scrapie groups were measured using one-way ANOVA followed by Tukey’s HSD (normally distributed data) or the Kruskal‒Wallis test followed by Dunn’s test (non-normally distributed). *p value < 0.05, **p value < 0.01, ^*p* value < 0.1 (tendency).

RT-qPCR was used to measure the relative expression of the eight miRNAs in the blood of healthy, preclinical and naturally affected scrapie sheep. As represented in Fig. 4, five of these miRNAs (miR-223-3p, miR-1271-5p, let-7f-5p, miR-186-5p and miR-425-5p) were significantly upregulated in the clinical scrapie sheep compared with the healthy animals.

### Replication of blood miRNA expression in the central nervous system

**Figure 5:**
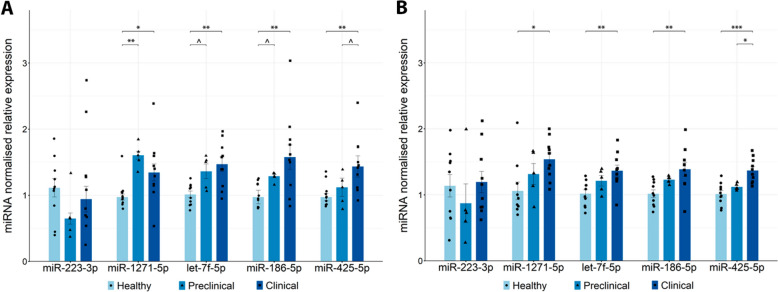
**Relative expression of significantly dysregulated miRNAs of interest in the blood measured by RT‒qPCR in the CNS, obex** (**A**) **and thalamus** (**B**). Grouped bar plot with overlaid scatter plot showing individual 2^−∆∆Ct^ values relative to the healthy group. The bars and error lines indicate the means of individual values and standard deviations or the medians and interquartile ranges for normally and non-normally distributed data, respectively. miRNA expression was normalised to the mean expression of miR-92a and miR-328 in the obex and to the mean expression of sn-U6, miR-320 and miR-328 in the thalamus. For each miRNA, significant differences between healthy (light blue and circles, *n* = 10) and preclinical (blue and triangles, *n* = 5) and clinical (dark blue and squares, *n* = 10) naturally affected scrapie groups were measured using one-way ANOVA followed by Tukey’s HSD (normally distributed data) or the Kruskal‒Wallis test followed by Dunn’s test (non-normally distributed). *p value < 0.05, **p value < 0.01, ***p value < 0.001, ^*p* value < 0.1 (tendency).

Since the miRNAs found in blood can originate from different tissues in the organism, we sought to investigate whether the five significantly dysregulated miRNAs in blood were also altered in the CNS. For this purpose, we analysed their expression by RT-qPCR in the most affected areas of the CNS in naturally affected scrapie sheep: the medulla oblongata at the level of the obex (Figure 5A) and thalamus (Figure 5B).

While we did not observe significant differences in the expression of miR-223 in either of the two areas of the CNS, the other four miRNAs presented significant increases in expression in clinical scrapie animals. Furthermore, in the obex, we observed very significant upregulation of miR-1271 (*p* value < 0.01) in preclinical animals and a close to significant tendency toward upregulation of let-7f and miR-186 (both *p* values < 0.1) in the preclinical stage of the disease compared with healthy animals.

### MiRNA expression strongly correlates with neuropathological features

**Figure 6:**
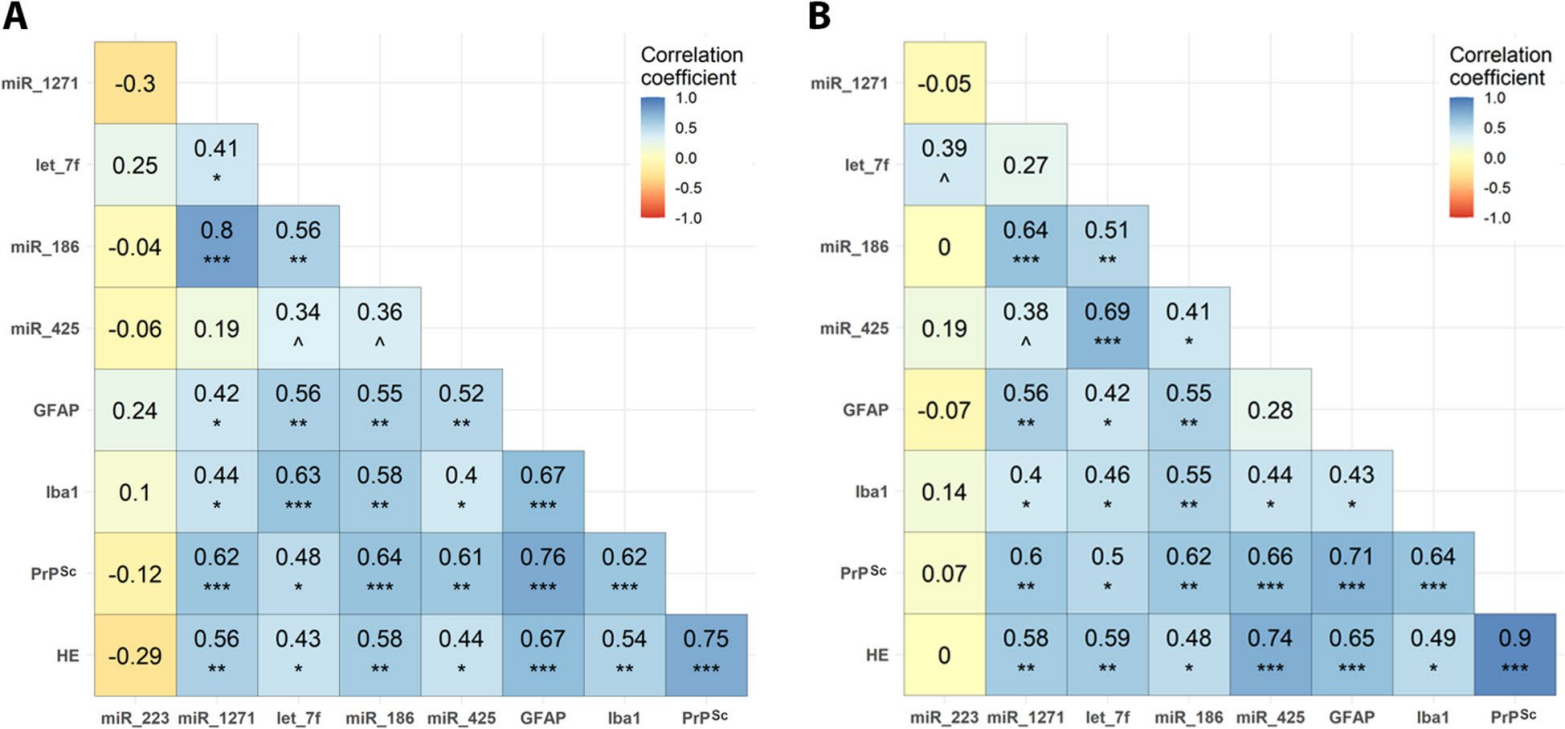
**Correlation matrix between the miRNAs of interest and prion neuropathology in the obex** (**A**) **and thalamus** (**B**). Spearman correlation coefficients and significance between the RT‒qPCR relative expression of five miRNAs in the CNS of healthy (*n* = 10) and preclinical (*n* = 5) and clinical (*n* = 10) naturally affected scrapie sheep and their neuropathological scores: PrP^Sc^ deposits (PrP^Sc^), spongiosis (HE), astrogliosis measured with glial fibrillary acidic protein (GFAP), and microgliosis measured using the ionised calcium-binding adaptor molecule 1 (Iba1). *p value < 0.05, **p value < 0.01, ***p value < 0.001, ^*p* value < 0.1 (tendency).

Having identified four miRNAs (miR-1271, let-7f, miR-186 and miR-425) significantly dysregulated in both blood and the two CNS areas studied, we sought to evaluate whether the miRNA expression levels were associated with the hallmark neuropathological features of prion diseases in the CNS: PrP^Sc^ deposits, spongiosis, astrogliosis and microgliosis. A correlation analysis was performed for each area between the RT-qPCR data from the miRNA expression in the CNS and the ImageJ scores of the neuropathological changes. Although the expression of miR-223 was not significantly altered in the CNS samples, it was included in the analysis because of its significantly altered expression in blood. As expected, miR-223 had no significant correlation with neuropathological features in either the obex (Figure 6A) or thalamus (Figure 6B). In contrast, the other four miRNAs showed very significant correlations with all or some of the neuropathological features examined here, in both areas, confirming their potential association with disease progression in the CNS.

### Significant miRNA target dysregulation in scrapie progression

Since it seems likely that miR-1271, let-7f, miR-186 and miR-425 could be directly related to prion pathology, they are most likely to affect the regulation of genes that play important roles in neurodegeneration. To explore this hypothesis further, we searched for predicted gene targets for these miRNAs. Using TarBase, miRDB and miRPath and confirming the homology of these miRNA sequences to those of humans, we selected four genes related to neurodegenerative pathways in which these four miRNAs are involved (Additional file 11): *KRAS*, *MDM2*, *CCND2* and *UBQLN2*. Additionally, we identified four other genes that are predicted to be targets of these miRNAs and have been found to be downregulated in previously published articles on scrapie-affected sheep [47, 48]: *DUSP1*, *IMMT*, *CLTC* and *PGK1*. Table 2 shows these target genes with their associated miRNAs and the pathways in which they are involved or their previously reported expression in scrapie sheep.

**Table 2:** **Gene targets of the significantly dysregulated miRNAs in the CNS.**

	Target gene	Gene name	miRNA^1^	Pathways^2^
Related to neurodegenerative pathways	*KRAS*	Kirsten rat sarcoma viral oncogene	let-7f, miR-1271	Autophagy-animal, apoptosis, pathways of neurodegeneration-multiple diseases, foxO signalling pathway
	*MDM2*	Mouse double minute 2	let-7f, miR-1271, miR-186, miR-425	p53 signalling pathway, foxO signalling pathway, ubiquitin mediated proteolysis
	*CCND2*	Cyclin D2	let-7f, miR-186	p53 signalling pathway, foxO signalling pathway
	*UBQLN2*	Ubiquilin 2	let-7f, miR-186, miR-425	Protein processing in endoplasmic reticulum

	Target gene	Gene name	miRNA^1^	Expression^3^
Previously reported downregulated	*DUSP1*	Dual specificity phosphatase 1	let-7f	(1) FC −2.4 *p* value < 0.05
	*IMMT*	MICOS complex subunit MIC60	let-7f	(2) FC −0.996 *p* value < 0.01
	*CLTC*	Clathrin heavy chain	let-7f, miR-1271, miR-186, miR-425	(2) FC −0.839 *p* value < 0.01
	*PGK1*	Phosphoglycerate kinase 1	let-7f, miR-1271, miR-186	(2) FC −0.554 *p* value < 0.05

^1^Abbreviated miRNA names for hsa-let-7f-5p, hsa-miR-1271-5p, hsa-miR-186-5p, and hsa-miR-425-5p

^2^Most relevant pathways related to neurodegenerative diseases

^3^Already published log_2_ fold change (FC) and *p* value of these genes, in article (1) [47] or article (2) [48]. In the case of article 1, it is gene expression, whereas in the case of article 2, the expression is at the protein level. Both compare scrapie-naturally affected clinical sheep to a healthy group

**Figure 7:**
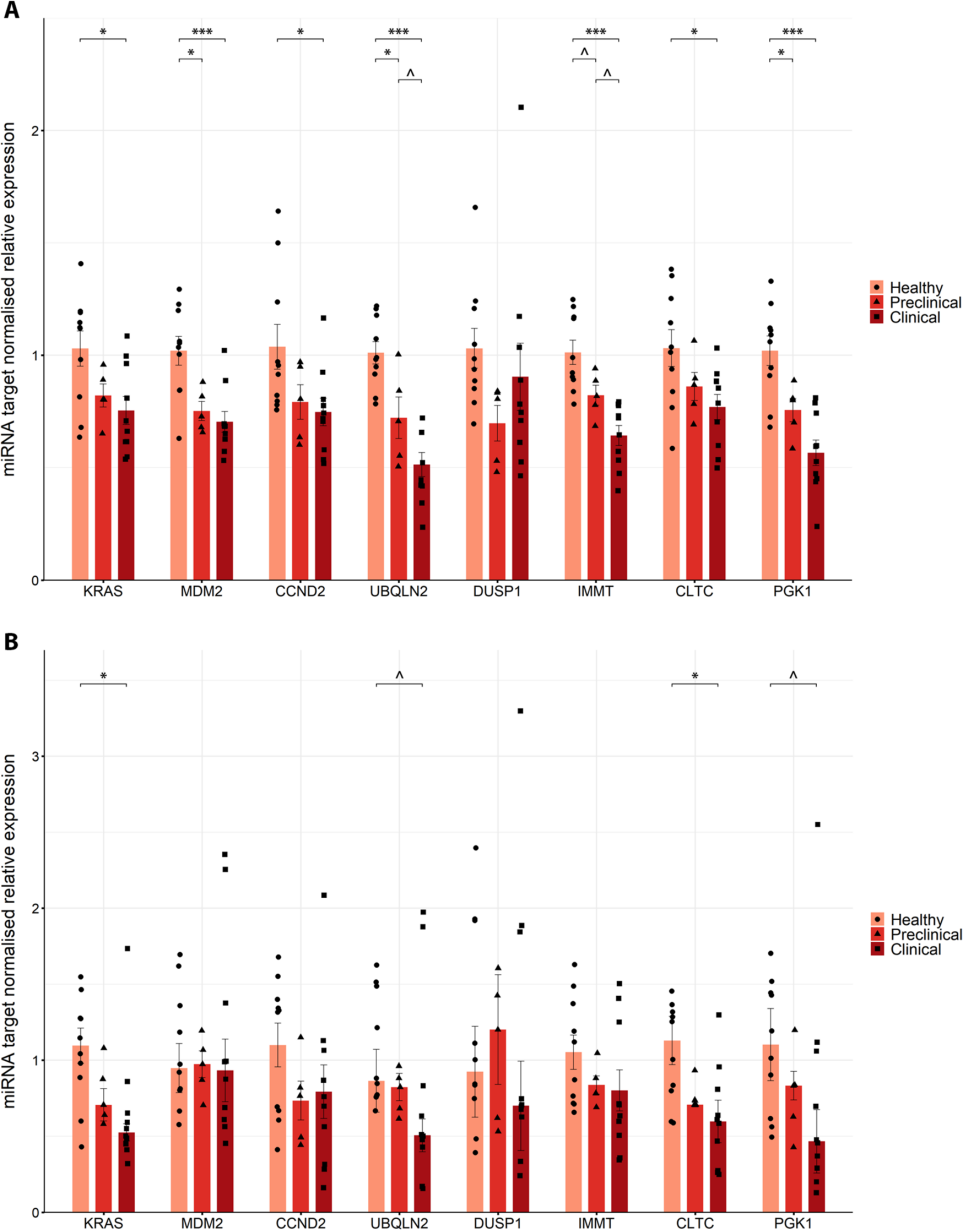
**Relative expression of selected miRNA gene targets measured by RT-qPCR in the CNS, obex** (**A**) **and thalamus** (**B**). Grouped bar plot with overlaid scatter plot showing individual 2^−∆∆Ct^ values relative to the healthy group. The bars and error lines indicate the means of individual values and standard deviations or the medians and interquartile ranges for normally and non-normally distributed data, respectively. Gene expression was normalised to the mean *G6PD* and *SDHA* in both the obex and thalamus. For each target gene, significant differences between healthy (light red and circles, *n* = 10) and preclinical (red and triangles, *n* = 5) and clinical (dark red and squares, *n* = 10) naturally affected scrapie groups were measured via one-way ANOVA followed by Tukey’s HSD (normally distributed data) or the Kruskal‒Wallis test followed by Dunn’s test (non-normally distributed). *p value < 0.05, ***p value < 0.001, ^*p* value < 0.1 (tendency).

To further explore whether miRNAs may regulate their target expression through mRNA degradation, we analysed the expression of the target genes we predicted from significantly upregulated miRNAs using RT-qPCR in the CNS, both the obex (Figure 7A) and the thalamus (Figure 7B). In the present study, all target genes except for *DUSP1* were found to be significantly downregulated in naturally affected scrapie sheep compared with healthy sheep, which is consistent with miRNA regulation. Notably, *MDM2*, *UBQLN2* and *PGK1* were also significantly downregulated in the preclinical stage group compared with the healthy stage group, whereas *IMMT* was nearly significantly decreased (*p* value < 0.1) in the preclinical stage group. In contrast, gene expression in the thalamus showed greater variability, and significant downregulation was detected only for *KRAS* and *CLTC* in clinical scrapie animals compared with healthy controls. *UBQLN2* and *PGK1* also tended to be downregulated in the clinical stage of disease.

## Discussion

The importance of miRNAs as biomarkers in body fluids for detecting disease presence or prognosis has increased in recent years. In this study, we explored circulating miRNAs in a natural model of prion disease, specifically sheep with classical scrapie, in which preclinical animals can be identified by rectal lymphoid biopsies performed in vivo. Using next-generation sequencing, we investigated for the first time differences in miRNA expression in blood from healthy animals and preclinical and clinical naturally affected classical scrapie sheep. Owing to the limited miRNA annotation for this species, we used the bioinformatic tool miRDeep2, which addresses this limitation by predicting novel and mature miRNAs on the basis of available annotations from other species. After statistical comparisons between groups, we identified one novel miRNA dysregulated in the preclinical stage and 126 miRNAs that were altered in the clinical stage of disease compared with healthy animals. Among these clinically dysregulated miRNAs, five were validated in blood by RT-qPCR, and four of them were also found to be altered in the CNS. These miRNAs presented expression levels that were significantly correlated with the typical neuropathological features of prion diseases: PrP^Sc^ deposits, spongiosis and gliosis.

Overall, our sequencing results indicate that the dysregulation of circulating miRNAs in the blood predominantly begins during the clinical stage of the disease. The minor differences found in the preclinical stage of the disease may partly reflect the limited number of preclinical animals used in this study. However, our analyses of miRNA expression in the CNS, particularly in the obex, revealed a tendency toward upregulation during the preclinical stage, suggesting early, although subtle, changes in the brain prior to the onset of clinical signs. This observation implies that miRNA changes in blood occur later than those in the CNS, potentially limiting their utility as circulating biomarkers for the preclinical stage. These results in a natural model of prion disease align with other studies in murine models, where minimal miRNA changes were observed in the CNS of preclinical animals [14, 15]. However, those studies were unable to validate their CNS sequencing findings in body fluids, such as plasma and cerebrospinal fluid. This discrepancy may suggest that the major differences in the CNS in terms of miRNA expression are not reflected in circulating fluids. In contrast, our reverse approach, beginning with blood sequencing and validating findings in the CNS, revealed that four of the five miRNAs altered in the blood were significantly dysregulated in the CNS. These findings indicate that most of the miRNA changes observed in the blood of scrapie animals likely originate from the CNS, further supporting the hypothesis that the CNS serves as a primary source for miRNA dysregulation in body fluids during the clinical stage of prion disease.

To further investigate the potential role of the dysregulated miRNAs identified in the clinical stage of the disease, we performed a KEGG pathway enrichment analysis. Several pathways related to neurodegeneration were among the top 20 significantly dysregulated pathways. Notably, we highlight autophagy, ubiquitin-mediated proteolysis, and protein processing in the endoplasmic reticulum pathway given their previously reported dysregulation in scrapie and other prion diseases [54, 55]. Interestingly, these pathways remained significant when the KEGG analysis was repeated using only the four miRNAs that were dysregulated in both the blood and the CNS. These findings suggest that the selection of miRNAs for validation by RT-qPCR was representative of the broader set of altered miRNAs.

Our finding of upregulated miR-1271 in the blood and CNS, particularly in the obex during the preclinical stage, may suggest its potential early role in disease progression. There is limited research on this miRNA in neurodegenerative diseases, and it has not been previously studied in prion diseases. However, upregulation of miR-1271 has been reported in the frontal cortex and cell models of AD, which is consistent with our results [56, 57].

In contrast, let-7f has been well researched and has been linked to neuroinflammation, apoptosis and oxidative stress [58, 59]. Interestingly, let-7f has been found to be upregulated in serum from CWD animals [60], which is in accordance with our findings on the upregulation of let-7f in both the blood and CNS from scrapie-affected animals, with a nearly significant tendency toward upregulation in the obex of preclinical sheep. Moreover, other miRNAs of the let-7 family have also been found to be upregulated in exosomes from prion-infected neuronal cells and in the brains of scrapie-infected mice and sCJD patients [14, 61, 62]. In contrast, let-7f often appears to be downregulated in other neurodegenerative disorders, such as AD, PD and ALS [63–65], indicating a rather specific and promising role for the let-7 family in prion diseases.

Interestingly, miR-186 has been researched for its potential role in regulating β-amyloid aggregation, a key feature of AD [66, 67], suggesting that it may similarly influence protein aggregation processes in prion disorders. In contrast to the upregulation of miR-186 shown in the present study, others have reported that miR-186 is downregulated in exosomes from prion-infected cells and serum from CWD cervids [60, 61]. However, a trend toward upregulation was found in the brains of scrapie-infected mice, similar to our results [62]. The overexpression of this miRNA has been linked to proapoptotic effects and reduced synaptic activity [68, 69]. Findings in AD, on the other hand, display high variability, with both up- and downregulation of miR-186 across different brain areas, indicating region-specific expression [70, 71].

Similarly, miR-425 has also been implicated in β-amyloid processing, promoting β-amyloid plaque formation [72, 73]. Although there are no previous studies of miR-425 in prion diseases, our significant finding of the upregulation of miR-425 in the blood and CNS of scrapie sheep, alongside its reported upregulation in AD blood and association with tau phosphorylation [65, 74], further supports its involvement in neurotoxic protein aggregation and neurodegeneration. Conversely, miR-425 has also been found to be downregulated in AD brains and PD cell models, linking its deficiency with enhanced neuroinflammation and necroptosis [73, 75].

Overall, as shown above, considerable variability exists across studies concerning miRNA expression. As revealed by others, specific miRNAs may have opposite expression levels depending on the disease [13]. Additionally, this variability may be further influenced by differences in disease models, tissue types and methodological approaches. Therefore, caution is needed when interpreting miRNA data across different studies, especially as expression patterns may reflect the unique pathophysiology of each disorder.

Here, we selected potential target genes of these four miRNAs from the most dysregulated KEGG pathways and previous scrapie research to provide insights into their potential role in the neuropathology of prion diseases and, more specifically, of scrapie. Although we cannot confirm that the target genes identified here are regulated by the miRNAs of interest that were dysregulated in our study, owing to the lack of direct experimental validation in our samples, it is worth noting that we used highly restrictive criteria to select potential targets from previously experimentally validated miRNA‒gene interactions in TarBase. Furthermore, our findings show that most of the target genes identified are downregulated, which is consistent with the expected result, since miRNAs generally act by repressing the expression of their target genes. Nonetheless, owing to the limited availability of sheep-specific genomic resources, human databases have been used to predict miRNA‒target interactions. Although seed sequence conservation between sheep and humans has been confirmed, interspecies differences may compromise the accuracy of these predictions. These findings further highlight the preliminary nature of our miRNA target results and reinforce the need for future functional validation experiments in ovine samples to confirm the functional relevance of these interactions. Interestingly, the expression of *UBQLN2* and *PGK1* was significantly lower in the obex of preclinical animals than in that of healthy controls. *UBQLN2*, which is known to be involved in the degradation of misfolded proteins through the ubiquitin‒proteasome system, is downregulated in AD and other neurodegenerative disorders, and mutations in this gene have been linked to ALS [76, 77]. Similarly, *PGK1* has been shown to promote autophagy and degrade pathological protein aggregates in AD [78]. While these two genes tend to be expressed only in the thalamus in the clinical stage, *KRAS* and *CLTC* downregulation was consistently significant in the clinical stage of disease in both CNS areas, and both genes have been previously reported to be downregulated in AD [79, 80]. Moreover, *CLTC* and *PGK1* protein expression has been previously reported to be downregulated in the CSF of scrapfish sheep [48]. Despite some interesting findings, it is important to note the marked disparity observed between the two CNS regions analysed, as well as the difficulty in identifying a clear cumulative regulatory effect between the validated miRNAs and their predicted target genes. CNS regions may harbour distinct, potentially numerous, dysregulated miRNAs not considered in this study, which may have synergistic or tissue-specific effects on gene expression. Given these limitations, more studies should be conducted to further explore the regional variability within the CNS, which may influence the extent of miRNA-mediated gene regulation in prion diseases.

Working with the naturally occurring disease in sheep may result in greater variability in the pathological stage among animals within the same group of diseases, thus potentially hiding differences in miRNA dysregulation. However, the significant correlations observed between miRNA expression and neuropathology levels seem to overcome this limitation, supporting notable changes throughout disease progression. Moreover, the use of naturally scrapie-affected animals poses an additional challenge in identifying individuals at the preclinical stage. This resulted in a limited sample size in this group, which may reduce the statistical robustness of our findings for early-stage disease changes. Therefore, these results should be interpreted with caution and confirmed in future studies involving a larger number of animals.

In conclusion, as the first study to explore miRNA expression in blood and CNS tissues in both preclinical and clinical scrapie-affected sheep, our findings reveal widespread miRNA dysregulation, with a key validated subset consisting of miR-1271, let-7f, miR-186 and miR-425, which showed significant upregulation in the blood of clinical animals and a tendency toward significantly increased expression in the most affected brain region of preclinical sheep, correlating positively with prion neuropathology. These findings suggest that although early-stage changes in miRNA expression may be challenging to detect, miRNA dysregulation may still serve as a valuable predictor of disease progression. Given the existing variability in miRNA expression, further research is needed to clarify the specific roles of the miRNAs validated here in prion diseases and to evaluate their potential role in disease progression.

## Supplementary Information

**Additional file 1. TaqMan probes used for RT-qPCR analysis of selected and potential housekeeping miRNAs in the blood and CNS**.

**Additional file 2. Raw Ct values (A, C, E) and RefFinder results (B, D, F) for the housekeeping miRNAs analysed in blood (A, B), obex (C, D) and thalamus (E, F) samples**. A, C and E, The means and standard deviations or medians and interquartile ranges for normally and non-normally distributed data, respectively, are shown for each group: healthy sheep (light blue circles, *n* = 10) and preclinical (blue triangles, *n* = 5) and clinical (dark blue squares, *n* = 10) naturally affected scrapie sheep. B, D and F, Overall stability rankings of candidate housekeeping miRNAs, which were calculated on the basis of the geometric means of the ranking values derived from multiple computational algorithms.

**Additional file 3. Primers used for RT-qPCR analysis of potential miRNA gene targets**.

**Additional file 4. Raw Ct values (A, C) and RefFinder results (B, D) for the housekeeping genes analysed in obex (A, B) and thalamus (C, D) samples**. A and C, Means and standard deviations or medians and interquartile ranges for normally and non-normally distributed data, respectively, are shown for each group: healthy sheep (light red circles, *n* = 10) and preclinical (red triangles, *n* = 5) and clinical (dark red squares, *n* = 10) naturally affected scrapie sheep. B and D, Overall stability rankings of candidate housekeeping genes, which are calculated on the basis of the geometric means of the ranking values derived from multiple computational algorithms.

**Additional file 5. Novel significantly dysregulated miRNAs in blood from preclinical scrapie sheep compared with healthy sheep**.

**Additional file 6. Predicted secondary structure of the preclinical putative novel miRNA precursor.** Compared with that of healthy controls, the stem‒loop structure of the precursor sequence of the novel miRNA was significantly dysregulated in the preclinical stage. Each nucleotide is color-coded, representing the base-pairing probability, where values range from 0 (low probability of being base-paired) to 1 (high probability of being base-paired).

**Additional file 7. Novel significantly dysregulated miRNAs in blood from clinical scrapie sheep compared with healthy sheep.**

**Additional file 8. Known significantly dysregulated miRNAs in blood from clinical scrapie sheep compared with healthy sheep.**

**Additional file 9. High similarity of novel significantly dysregulated miRNAs to previously reported miRNAs in ruminant studies.**

**Additional file 10. KEGG pathways involving significantly dysregulated (adjusted p value < 0.01) miRNAs in blood.**

**Additional file 11. KEGG pathways involving the significantly dysregulated (adjusted** ***p*** **value < 0.01) miRNAs in the CNS.**

## Data Availability

The raw fastq.gz sequencing files and normalised read counts have been deposited in the NCBI Gene Expression Omnibus (GEO) and are accessible through GEO series accession number GSE287730.

## Abbreviations

AD: Alzheimer’s disease
adj *p* value: adjusted *p* value
ALS: amyotrophic lateral sclerosis
bp: base pairs
cDNA: complementary DNA
CNS: central nervous system
CSF: cerebrospinal fluid
Ct: cycle threshold
CWD: chronic wasting disease
FC: fold change
FDR: false discovery rate
GFAP: glial fibrillary acidic protein
HE: haematoxylin and eosin
Iba1: ionised calcium-binding adaptor molecule 1
IHC: immunohistochemistry
KEGG: Kyoto Encyclopedia of Genes and Genomes
MFE: minimum folding energy
miRNAs: microRNAs
mRNA: messenger RNA
PCA: principal component analysis
PD: Parkinson’s disease
PrP^C^: cellular prion protein
PrP^Sc^: pathological prion protein
RIN: RNA integrity number
RISC: RNA-induced silencing complex
RT: room temperature
RT-qPCR: real-time quantitative PCR
sCJD: sporadic Creutzfeldt‒Jakob disease
TSEs: transmissible spongiform encephalopathies
