# Supplementary material for: Dysregulated microRNAs in blood correlate with central nervous system neuropathology of prion disease
Source: Vet Res. 2025 Jul 1;56:132. doi: 10.1186/s13567-025-01566-0 (PMC12220440; doi:10.1186/s13567-025-01566-0)
Supplement: Supplementary file 1 — Additional file 1. TaqMan probes used for RT-qPCR analysis of selected and potential housekeeping miRNAs in the blood and CNS. [file 13567_2025_1566_MOESM1_ESM.docx]

**Additional file 1.** **TaqMan probes used for RT-qPCR analysis of selected and potential housekeeping miRNAs in blood and CNS.**

| Assay Name | Assay ID | Selected miRNA / potential housekeeping |
| --- | --- | --- |
| hsa-miR-92a | 000431 | Potential housekeeping |
| hsa-miR-222 | 000525 | Potential housekeeping |
| hsa-miR-320 | 002277 | Potential housekeeping |
| hsa-miR-328 | 000543 | Potential housekeeping |
| U6 snRNA | 001973 | Potential housekeeping |
| hsa-miR-223 | 002295 | Selected miRNA |
| hsa-miR-425-5p | 001516 | Selected miRNA |
| mmu-let-7f | 001769 | Selected miRNA |
| hsa-miR-186 | 002285 | Selected miRNA |
| chi-miR-1271-5p | 477599_mat | Selected miRNA |
| bta-miR-30e-5p | 007791_mat | Selected miRNA |
| cgr-miR-140-3p | 471823_mat | Selected miRNA |
| cfa-miR-199 | 004405_mat | Selected miRNA |
