# Supplementary figures and images for: Dysregulated microRNAs in blood correlate with central nervous system neuropathology of prion disease
Source: Vet Res. 2025 Jul 1;56:132. doi: 10.1186/s13567-025-01566-0 (PMC12220440; doi:10.1186/s13567-025-01566-0)

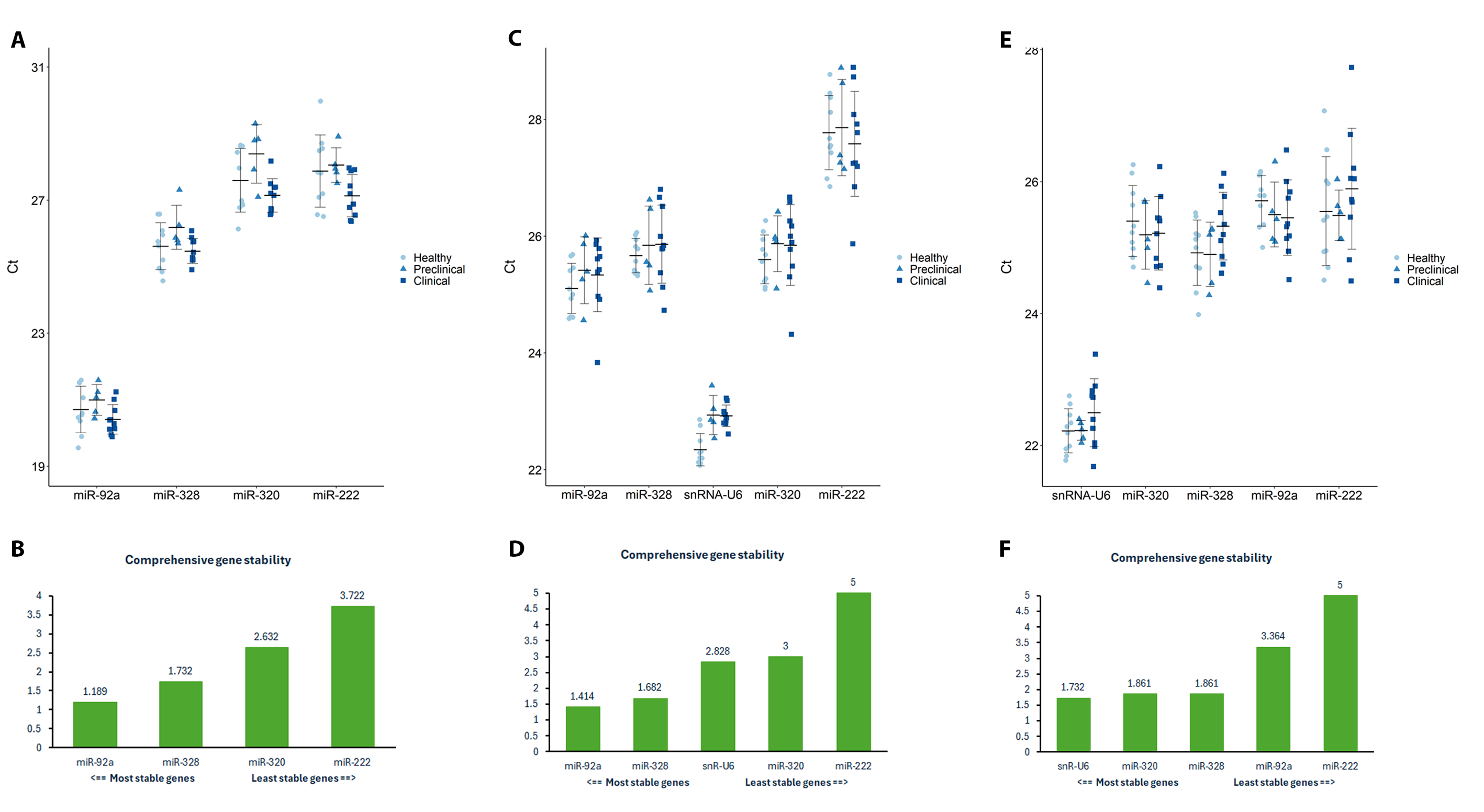

Supplement: Supplementary file 2 — Additional file 2. Raw Ct values (A, C, E) and RefFinder results (B, D, F) for the housekeeping miRNAs analysed in blood (A, B), obex (C, D) and thalamus (E, F) samples. A, C and E, The means and standard deviations or medians and interquartile ranges for normally and non-normally distributed data, respectively, are shown for each group: healthy sheep (light blue circles, n = 10) and preclinical (blue triangles, n = 5) and clinical (dark blue squares, n = 10) naturally affected scrapie sheep. B, D and F, Overall stability rankings of candidate housekeeping miRNAs, which were calculated on the basis of the geometric means of the ranking values derived from multiple computational algorithms. [file 13567_2025_1566_MOESM2_ESM.tif]

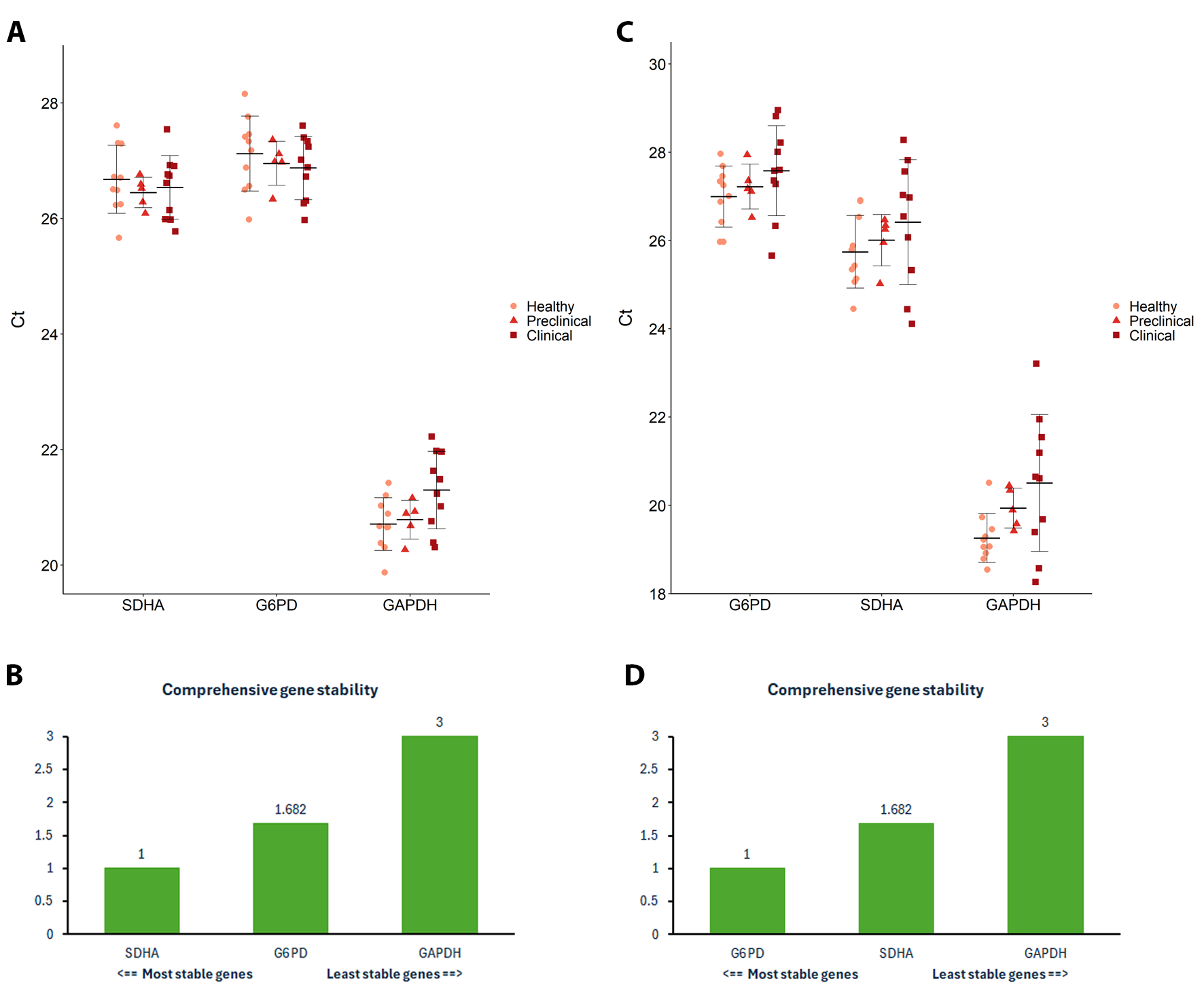

Supplement: Supplementary file 4 — Additional file 4. Raw Ct values (A, C) and RefFinder results (B, D) for the housekeeping genes analysed in obex (A, B) and thalamus (C, D) samples. A and C, Means and standard deviations or medians and interquartile ranges for normally and non-normally distributed data, respectively, are shown for each group: healthy sheep (light red circles, n = 10) and preclinical (red triangles, n = 5) and clinical (dark red squares, n = 10) naturally affected scrapie sheep. B and D, Overall stability rankings of candidate housekeeping genes, which are calculated on the basis of the geometric means of the ranking values derived from multiple computational algorithms. [file 13567_2025_1566_MOESM4_ESM.tif]

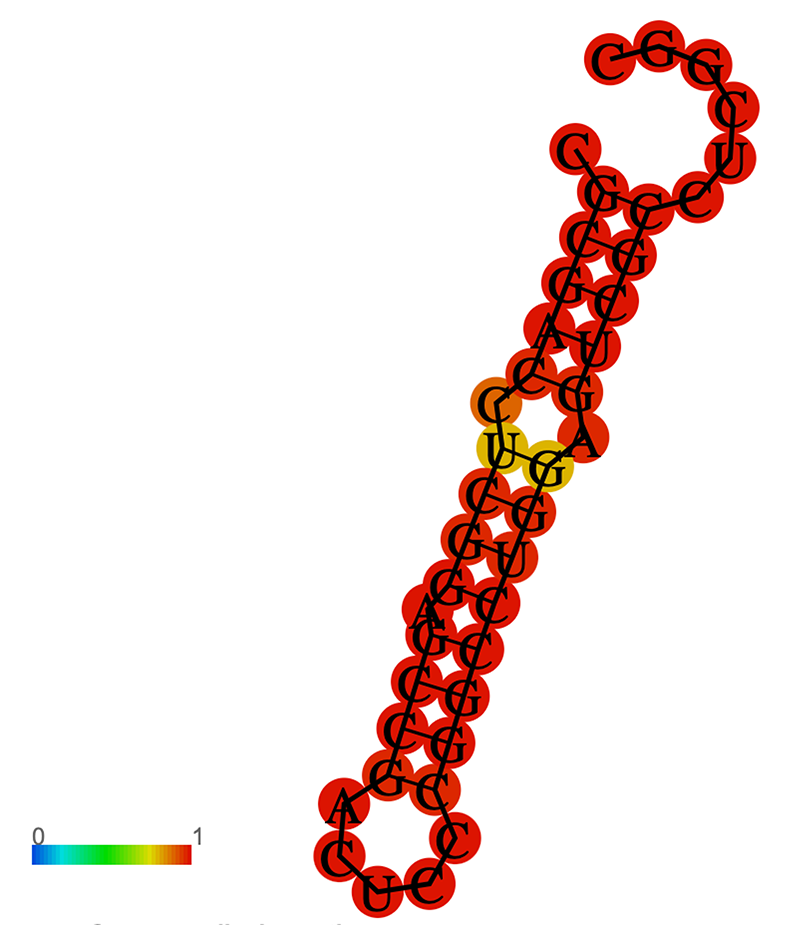

Supplement: Supplementary file 6 — Additional file 6. Predicted secondary structure of the preclinical putative novel miRNA precursor. Compared with that of healthy controls, the stem‒loop structure of the precursor sequence of the novel miRNA was significantly dysregulated in the preclinical stage. Each nucleotide is color-coded, representing the base-pairing probability, where values range from 0 (low probability of being base-paired) to 1 (high probability of being base-paired). [file 13567_2025_1566_MOESM6_ESM.tif]
