# Supplementary material for: Dysregulated microRNAs in blood correlate with central nervous system neuropathology of prion disease
Source: Vet Res. 2025 Jul 1;56:132. doi: 10.1186/s13567-025-01566-0 (PMC12220440; doi:10.1186/s13567-025-01566-0)
Supplement: Supplementary file 3 — Additional file 3. Primers used for RT-qPCR analysis of potential miRNA gene targets. [file 13567_2025_1566_MOESM3_ESM.docx]

**Additional file 3.** **Primers used for RT-qPCR analysis of potential miRNA gene targets**.

| **Gene** | **Forward (F) and reverse (R) primer sequences (5’🡪3’)** | **Concentration (nM)** | **Amplicon length (bp)** | **GenBank accession number** |
| --- | --- | --- | --- | --- |
| *GAPDH*^1^ | F: TCCATGACCACTTTGGCATCGT  R: GTCTTCTGGGTGGCAGTGA | 900 | 80 | NM_001190390.1 |
| *G6PD*^1^ | F: TGACCTATGGCAACCGATACAA  R: CCGCAAAAGACATCCAGGAT | 300 | 76 | NM_001093780.1 |
| *SDHA*^1^ | F: CATCCACTACATGACGGAGCA  R: ATCTTGCCATCTTCAGTTCTGCTA | 300 | 90 | XM_027980212.2 |
| *KRAS* | F: CAAGAGTGCCTTGACGATACAG  R: CCAAGAGACAGGTTTCTCCATC | 500 | 116 | XM_027967805.3 |
| *MDM2* | F: GGCAGGCGAGAGTGATACAG  R: AGGTGGAAGGGGAGGATTCA | 300 | 106 | XM_012174596.5 |
| *CCND2* | F: GCAAAGATCACCAACACGGAT  R: CCAGCTCATCCTCCGACTT | 300 | 130 | NM_001127290.1 |
| *UBQLN2* | F: CCACGAGCCCAATATCGGAA  R: CCCATTGCGTTGAGCTGTTC | 300 | 142 | XM_004022044.5 |
| *DUSP1* | F: TGTCCCAACCATTTCGAGGG  R: AAACACCCTTCCACCAGCAT | 500 | 138 | XM_004016868.5 |
| *IMMT* | F: AGGAAAAGCGGGCATTTGAC  R: TCATTCTCCATGGCGTCTCTG | 300 | 115 | XM_004005886.5 |
| *CLTC* | F: TGCTATGATCCTGAGCGAGTC  R: GCACCAAATCGTGGACAAAG | 500 | 109 | XM_060395124.1 |
| *PGK1* | F: GCTGACAAGAATGGCGTGAA  R: CTCAGGACCACAGTCCAACC | 400 | 141 | NM_001142516.1 |

^1^ Primer sequences previously described by Lyahyai et al. [52].
