# Supplementary material for: Dysregulated microRNAs in blood correlate with central nervous system neuropathology of prion disease
Source: Vet Res. 2025 Jul 1;56:132. doi: 10.1186/s13567-025-01566-0 (PMC12220440; doi:10.1186/s13567-025-01566-0)
Supplement: Supplementary file 8 — Additional file 8. Known significantly dysregulated miRNAs in blood from clinical scrapie sheep compared with healthy sheep. [file 13567_2025_1566_MOESM8_ESM.docx]

**Additional file 8.** **Known significantly dysregulated miRNAs in blood from clinical scrapie sheep compared to healthy sheep.**

| **Upregulated miRNAs** | | | | | | |
| --- | --- | --- | --- | --- | --- | --- |
| **miRNA** | **Log_2_FC^1^** | ***p*-value** | **adj *p-*value^2^** | **Counts_C^3^** | **Counts_H^3^** |  |
| miR-223 | 2.30 | 1.42E-09 | 9.46E-07 | 576 ± 360 | 125 ± 91 |  |
| miR-425 | 0.94 | 6.34E-09 | 2.11E-06 | 1704 ± 392 | 957 ± 128 |  |
| let-7f | 1.76 | 1.55E-08 | 2.59E-06 | 444 ± 304 | 156 ± 44 |  |
| miR-30c | 1.45 | 1.31E-08 | 2.59E-06 | 5348 ± 3321 | 1787 ± 723 |  |
| miR-149 | 2.53 | 1.15E-07 | 1.27E-05 | 29 ± 41 | 5 ± 5 |  |
| miR-374b | 3.54 | 1.75E-07 | 1.67E-05 | 14 ± 28 | 3 ± 4 |  |
| miR-186 | 1.53 | 3.17E-07 | 2.64E-05 | 2577 ± 2234 | 1267 ± 161 |  |
| miR-23a | 1.97 | 4.44E-07 | 3.29E-05 | 115 ± 79 | 23 ± 21 |  |
| miR-21 | 2.49 | 6.81E-07 | 4.54E-05 | 211 ± 170 | 51 ± 33 |  |
| miR-30b | 2.03 | 7.73E-07 | 4.69E-05 | 42 ± 61 | 18 ± 10 |  |
| miR-1271 | 1.56 | 2.14E-06 | 1.19E-04 | 285 ± 156 | 116 ± 37 |  |
| miR-25 | 0.83 | 5.01E-06 | 2.57E-04 | 50155 ± 20146 | 29919 ± 8992 |  |
| miR-194 | 1.42 | 6.41E-06 | 3.06E-04 | 87 ± 47 | 41 ± 13 |  |
| miR-30e | 1.72 | 7.93E-06 | 3.52E-04 | 358 ± 267 | 208 ± 99 |  |
| miR-28 | 1.41 | 9.87E-06 | 4.11E-04 | 83 ± 62 | 39 ± 18 |  |
| miR-140 | 1.07 | 1.26E-05 | 4.42E-04 | 3556 ± 1721 | 2059 ± 499 |  |
| miR-10a | 1.85 | 1.78E-05 | 5.75E-04 | 248 ± 134 | 125 ± 96 |  |
| miR-192 | 1.69 | 2.68E-05 | 7.64E-04 | 201 ± 110 | 117 ± 21 |  |
| miR-99a | 1.66 | 2.91E-05 | 7.64E-04 | 22577 ± 18224 | 11672 ± 2842 |  |
| miR-26b | 1.60 | 2.91E-05 | 7.64E-04 | 231 ± 297 | 107 ± 76 |  |
| miR-1388 | 0.99 | 3.46E-05 | 8.54E-04 | 122 ± 69 | 63 ± 23 |  |
| miR-199a | 1.43 | 4.42E-05 | 9.51E-04 | 191 ± 161 | 116 ± 52 |  |
| miR-19b | 2.74 | 4.76E-05 | 9.92E-04 | 12 ± 19 | 4 ± 2 |  |
| miR-6119 | 2.37 | 5.11E-05 | 0.001 | 21 ± 36 | 6 ± 3 |  |
| miR-30f | 1.33 | 5.80E-05 | 0.001 | 28 ± 16 | 9 ± 7 |  |
| miR-7 | 2.57 | 8.25E-05 | 0.001 | 143 ± 214 | 27 ± 54 |  |
| miR-29a | 1.22 | 8.84E-05 | 0.001 | 159 ± 17 | 87 ± 31 |  |
| miR-128 | 0.97 | 1.08E-04 | 0.002 | 20700 ± 7355 | 13095 ± 3792 |  |
| miR-128-2 | 1.02 | 1.53E-04 | 0.002 | 1945 ± 843 | 1300 ± 325 |  |
| miR-151 | 1.22 | 2.43E-04 | 0.003 | 5263 ± 2336 | 3085 ± 1593 |  |
| miR-98 | 1.73 | 2.71E-04 | 0.003 | 88 ± 103 | 32 ± 33 |  |
| miR-16a | 1.14 | 3.22E-04 | 0.004 | 19781 ± 15179 | 8869 ± 6286 |  |
| miR-340 | 1.34 | 4.19E-04 | 0.004 | 103 ± 111 | 57 ± 44 |  |
| miR-345 | 1.46 | 4.54E-04 | 0.005 | 10 ± 7 | 4 ± 3 |  |
| miR-155 | 1.19 | 4.62E-04 | 0.005 | 75 ± 74 | 44 ± 29 |  |
| miR-421 | 1.69 | 6.13E-04 | 0.006 | 10 ± 2 | 5 ± 6 |  |
| miR-148a | 1.90 | 6.46E-04 | 0.006 | 306 ± 357 | 168 ± 67 |  |
| miR-23b | 1.15 | 6.86E-04 | 0.006 | 107 ± 59 | 56 ± 33 |  |
| miR-100 | 1.34 | 7.85E-04 | 0.007 | 60 ± 64 | 37 ± 7 |  |
| miR-664b | 1.54 | 9.49E-04 | 0.009 | 12 ± 8 | 4 ± 2 |  |
| miR-2284w | 1.04 | 9.81E-04 | 0.009 | 131 ± 107 | 76 ± 20 |  |
| miR-374a | 3.00 | 0.001 | 0.009 | 6 ± 10 | 1 ± 3 |  |
| miR-769 | 0.93 | 0.001 | 0.010 | 37 ± 40 | 28 ± 5 |  |
| let-7g | 1.28 | 0.002 | 0.011 | 1776 ± 1099 | 837 ± 774 |  |
| miR-15b | 0.82 | 0.002 | 0.012 | 1029 ± 300 | 559 ± 387 |  |
| miR-224 | 0.99 | 0.002 | 0.013 | 22 ± 8 | 13 ± 3 |  |
| miR-22 | 0.83 | 0.002 | 0.013 | 276 ± 57 | 200 ± 97 |  |
| miR-196b | 1.34 | 0.002 | 0.017 | 101 ± 80 | 29 ± 37 |  |
| miR-24-2 | 0.82 | 0.003 | 0.017 | 158 ± 71 | 116 ± 55 |  |
| miR-107 | 1.08 | 0.003 | 0.017 | 151 ± 143 | 111 ± 46 |  |
| miR-16b | 0.84 | 0.003 | 0.018 | 15451 ± 7212 | 8419 ± 7569 |  |
| miR-27a | 0.98 | 0.003 | 0.019 | 59 ± 41 | 45 ± 21 |  |
| miR-20a | 1.93 | 0.003 | 0.020 | 27 ± 49 | 9 ± 27 |  |
| miR-27b | 1.08 | 0.003 | 0.021 | 246 ± 161 | 163 ± 68 |  |
| miR-15a | 1.93 | 0.004 | 0.023 | 9 ± 11 | 6 ± 6 |  |
| miR-10b | 1.84 | 0.005 | 0.028 | 6 ± 12 | 4 ± 2 |  |
| miR-142 | 1.40 | 0.007 | 0.034 | 939 ± 1585 | 611 ± 438 |  |
| miR-2284x | 1.12 | 0.007 | 0.034 | 89 ± 82 | 53 ± 37 |  |
| miR-335 | 1.01 | 0.008 | 0.037 | 310 ± 170 | 143 ± 97 |  |
| let-7a | 0.92 | 0.009 | 0.043 | 6259 ± 5231 | 2962 ± 2509 |  |
| miR-495 | 1.33 | 0.010 | 0.046 | 8 ± 5 | 4 ± 4 |  |
| miR-660 | 0.89 | 0.010 | 0.046 | 27 ± 20 | 21 ± 10 |  |
| **Downregulated miRNAs** | | | | | | |
| **miRNA** | **Log_2_FC^1^** | ***p*-value** | **adj *p-*value^2^** | **Counts_C^3^** | **Counts_H^3^** |  |
| miR-7865 | -1.15 | 1.90345E-05 | 5.77E-04 | 159 ± 81 | 371 ± 143 |  |
| miR-370 | -1.12 | 0.005 | 0.027 | 13 ± 8 | 30 ± 14 |  |
| miR-197 | -0.88 | 0.010 | 0.044 | 9136 ± 7306 | 15029 ± 3417 |  |

^1^Log_2_FC: log_2_ fold change.

^2^adj *p*-value: adjusted *p*-value using Benjamini-Hochberg false discovery rate correction.

^3^Counts: median small RNA sequencing read counts in all samples in each group (C: clinical stage, H: healthy controls) ± interquartile range.
