# Supplementary material for: Dysregulated microRNAs in blood correlate with central nervous system neuropathology of prion disease
Source: Vet Res. 2025 Jul 1;56:132. doi: 10.1186/s13567-025-01566-0 (PMC12220440; doi:10.1186/s13567-025-01566-0)
Supplement: Supplementary file 10 — Additional file 10. KEGG pathways involving significantly dysregulated (adjusted p value < 0.01) miRNAs in blood. [file 13567_2025_1566_MOESM10_ESM.docx]

**Additional file 10.** **KEGG pathways involving significantly dysregulated (adjusted *p-*value < 0.01) miRNAs in blood.**

| **KEGG pathway** | **adj *p*-value^1^** | **Target genes (n)** | **miRNAs (n)** |
| --- | --- | --- | --- |
| Ubiquitin mediated proteolysis | 3.64E-19 | 124 | 56 |
| Pathways in cancer | 9.90E-17 | 375 | 56 |
| Shigellosis | 1.61E-15 | 200 | 56 |
| Autophagy - animal | 1.24E-14 | 120 | 55 |
| Cell cycle | 6.24E-13 | 106 | 56 |
| Proteoglycans in cancer | 2.56E-12 | 163 | 57 |
| FoxO signalling pathway | 4.83E-12 | 111 | 56 |
| Renal cell carcinoma | 2.31E-11 | 63 | 55 |
| Amyotrophic lateral sclerosis | 1.03E-10 | 270 | 56 |
| Protein processing in endoplasmic reticulum | 1.11E-10 | 143 | 55 |
| Salmonella infection | 2.96E-10 | 192 | 56 |
| Pathways of neurodegeneration - multiple diseases | 6.36E-10 | 341 | 56 |
| Chronic myeloid leukemia | 1.21E-09 | 67 | 56 |
| Focal adhesion | 2.71E-09 | 151 | 56 |
| HIF-1 signalling pathway | 3.95E-09 | 88 | 55 |
| Huntington disease | 5.25E-09 | 224 | 56 |
| Colorectal cancer | 5.35E-09 | 72 | 56 |
| p53 signalling pathway | 7.47E-09 | 63 | 56 |
| Neurotrophin signalling pathway | 7.47E-09 | 95 | 55 |
| Prostate cancer | 9.96E-09 | 80 | 55 |
| Alzheimer disease | 1.13E-08 | 272 | 56 |
| Parkinson disease | 1.78E-08 | 189 | 56 |
| Axon guidance | 2.11E-08 | 132 | 55 |
| Rap1 signalling pathway | 3.81E-08 | 148 | 56 |
| Thermogenesis | 4.80E-08 | 170 | 56 |
| Yersinia infection | 6.93E-08 | 107 | 55 |
| Apelin signalling pathway | 1.40E-07 | 102 | 56 |
| ErbB signalling pathway | 1.47E-07 | 68 | 56 |
| Small cell lung cancer | 1.47E-07 | 77 | 55 |
| Hepatocellular carcinoma | 1.80E-07 | 124 | 55 |
| RNA transport | 4.01E-07 | 136 | 55 |
| Fluid shear stress and atherosclerosis | 4.86E-07 | 106 | 56 |
| Regulation of actin cytoskeleton | 5.97E-07 | 150 | 56 |
| Hepatitis B | 8.39E-07 | 122 | 56 |
| Endocytosis | 9.52E-07 | 199 | 56 |
| TGF-beta signalling pathway | 9.97E-07 | 77 | 56 |
| MAPK signalling pathway | 9.97E-07 | 209 | 56 |
| mRNA surveillance pathway | 1.07E-06 | 80 | 55 |
| Alcoholism | 1.19E-06 | 132 | 56 |
| AGE-RAGE signalling pathway in diabetic complications | 1.43E-06 | 84 | 55 |
| Hippo signalling pathway | 4.94E-06 | 112 | 56 |
| Transcriptional misregulation in cancer | 6.70E-06 | 136 | 56 |
| Pathogenic Escherichia coli infection | 7.47E-06 | 145 | 56 |
| Glioma | 8.19E-06 | 60 | 55 |
| Insulin signalling pathway | 1.53E-05 | 104 | 55 |
| PI3K-Akt signalling pathway | 2.12E-05 | 227 | 56 |
| Thyroid hormone signalling pathway | 2.37E-05 | 94 | 55 |
| Apoptosis | 2.76E-05 | 102 | 55 |
| TNF signalling pathway | 3.36E-05 | 90 | 55 |
| Tight junction | 5.07E-05 | 119 | 55 |
| Signalling pathways regulating pluripotency of stem cells | 5.09E-05 | 104 | 56 |
| Metabolic pathways | 6.03E-05 | 887 | 56 |
| Oocyte meiosis | 1.25E-04 | 90 | 55 |
| Growth hormone synthesis, secretion and action | 1.30E-04 | 87 | 56 |
| Hepatitis C | 1.60E-04 | 112 | 55 |
| Progesterone-mediated oocyte maturation | 1.60E-04 | 72 | 55 |
| Oxytocin signalling pathway | 1.72E-04 | 105 | 56 |
| Endocrine resistance | 1.85E-04 | 80 | 56 |
| Fc gamma R-mediated phagocytosis | 1.85E-04 | 70 | 55 |
| Non-small cell lung cancer | 1.86E-04 | 58 | 55 |
| Platinum drug resistance | 2.63E-04 | 54 | 56 |
| Relaxin signalling pathway | 2.77E-04 | 91 | 56 |
| Sphingolipid signalling pathway | 2.90E-04 | 88 | 56 |
| GnRH signalling pathway | 2.90E-04 | 67 | 55 |
| Chagas disease | 3.16E-04 | 78 | 56 |
| Neutrophil extracellular trap formation | 4.36E-04 | 128 | 56 |
| Necroptosis | 6.22E-04 | 110 | 55 |
| cGMP-PKG signalling pathway | 8.47E-04 | 110 | 56 |
| Melanoma | 9.53E-04 | 53 | 56 |
| Breast cancer | 9.53E-04 | 103 | 56 |
| Viral carcinogenesis | 0.001 | 159 | 56 |
| Cellular senescence | 0.002 | 133 | 57 |
| Ras signalling pathway | 0.002 | 145 | 56 |
| Gastric cancer | 0.002 | 101 | 56 |
| Prion disease | 0.002 | 193 | 56 |
| Wnt signalling pathway | 0.002 | 107 | 56 |
| Human papillomavirus infection | 0.002 | 233 | 55 |
| Prolactin signalling pathway | 0.002 | 50 | 55 |
| Choline metabolism in cancer | 0.003 | 69 | 55 |
| Kaposi sarcoma-associated herpesvirus infection | 0.004 | 145 | 56 |
| Dopaminergic synapse | 0.004 | 89 | 55 |
| Platelet activation | 0.004 | 85 | 55 |
| Cushing syndrome | 0.007 | 106 | 55 |
| Phospholipase D signalling pathway | 0.007 | 96 | 55 |
| Oxidative phosphorylation | 0.009 | 95 | 55 |

^1^adj *p*-value: adjusted *p*-value using Benjamini-Hochberg false discovery rate correction.
