# Supplementary material for: Dysregulated microRNAs in blood correlate with central nervous system neuropathology of prion disease
Source: Vet Res. 2025 Jul 1;56:132. doi: 10.1186/s13567-025-01566-0 (PMC12220440; doi:10.1186/s13567-025-01566-0)
Supplement: Supplementary file 11 — Additional file 11. KEGG pathways involving the significantly dysregulated (adjusted p value < 0.01) miRNAs in the CNS. [file 13567_2025_1566_MOESM11_ESM.docx]

**Additional file 11.** **KEGG pathways involving the significantly dysregulated (adjusted *p-*value < 0.01) miRNAs in the CNS.**

| **KEGG pathway** | **adj *p-*value^1^** | **Target genes (n)** | **miRNAs (n)** |
| --- | --- | --- | --- |
| Proteoglycans in cancer | 1.84E-07 | 78 | 4 |
| p53 signalling pathway | 1.84E-07 | 37 | 4 |
| Autophagy - animal | 4.08E-07 | 57 | 4 |
| FoxO signalling pathway | 2.78E-06 | 53 | 4 |
| Cell cycle | 2.93E-06 | 50 | 4 |
| Spinocerebellar ataxia | 3.50E-06 | 54 | 4 |
| Protein processing in endoplasmic reticulum | 6.23E-06 | 66 | 4 |
| AMPK signalling pathway | 6.94E-06 | 49 | 4 |
| Longevity regulating pathway | 6.99E-06 | 42 | 4 |
| Renal cell carcinoma | 1.52E-05 | 31 | 4 |
| Oocyte meiosis | 1.52E-05 | 49 | 4 |
| Ubiquitin mediated proteolysis | 1.52E-05 | 51 | 4 |
| Huntington disease | 1.57E-05 | 99 | 4 |
| Hippo signalling pathway | 2.42E-05 | 56 | 4 |
| Prostate cancer | 3.30E-05 | 39 | 4 |
| Long-term potentiation | 5.76E-05 | 30 | 4 |
| Adherens junction | 7.63E-05 | 32 | 4 |
| Alzheimer disease | 1.05E-04 | 115 | 4 |
| Thyroid hormone signalling pathway | 1.13E-04 | 47 | 4 |
| Pathways of neurodegeneration - multiple diseases | 1.43E-04 | 139 | 4 |
| Amyotrophic lateral sclerosis | 1.54E-04 | 110 | 4 |
| Apelin signalling pathway | 1.88E-04 | 47 | 4 |
| Transcriptional misregulation in cancer | 2.07E-04 | 63 | 4 |
| MAPK signalling pathway | 2.75E-04 | 91 | 4 |
| AGE-RAGE signalling pathway in diabetic complications | 2.75E-04 | 40 | 4 |
| Shigellosis | 2.75E-04 | 77 | 4 |
| Focal adhesion | 2.81E-04 | 64 | 4 |
| Hepatitis B | 3.46E-04 | 55 | 4 |
| mTOR signalling pathway | 3.46E-04 | 55 | 4 |
| Glioma | 3.84E-04 | 30 | 4 |
| Salmonella infection | 4.26E-04 | 78 | 4 |
| Pathways in cancer | 4.32E-04 | 139 | 4 |
| TGF-beta signalling pathway | 4.90E-04 | 36 | 4 |
| Small cell lung cancer | 5.84E-04 | 35 | 4 |
| Neurotrophin signalling pathway | 5.86E-04 | 41 | 4 |
| HIF-1 signalling pathway | 5.86E-04 | 38 | 4 |
| Regulation of actin cytoskeleton | 5.86E-04 | 65 | 4 |
| Phosphatidylinositol signalling system | 6.43E-04 | 35 | 4 |
| Fluid shear stress and atherosclerosis | 6.43E-04 | 47 | 4 |
| Platinum drug resistance | 7.41E-04 | 28 | 4 |
| Chronic myeloid leukaemia | 7.75E-04 | 29 | 4 |
| Signalling pathways regulating pluripotency of stem cells | 9.84E-04 | 48 | 4 |
| Hepatitis C | 1.00E-03 | 52 | 4 |
| Colorectal cancer | 1.00E-03 | 31 | 4 |
| Parkinson disease | 1.00E-03 | 77 | 4 |
| Oxytocin signalling pathway | 0.001 | 49 | 4 |
| Rap1 signalling pathway | 0.001 | 61 | 4 |
| PI3K-Akt signalling pathway | 0.001 | 96 | 4 |
| Longevity regulating pathway - multiple species | 0.002 | 28 | 4 |
| Measles | 0.002 | 48 | 4 |
| Insulin signalling pathway | 0.002 | 46 | 4 |
| Cellular senescence | 0.002 | 61 | 4 |
| Growth hormone synthesis, secretion and action | 0.002 | 40 | 4 |
| N-Glycan biosynthesis | 0.003 | 20 | 4 |
| Apoptosis | 0.003 | 45 | 4 |
| Pancreatic cancer | 0.003 | 27 | 4 |
| Glucagon signalling pathway | 0.003 | 36 | 4 |
| ErbB signalling pathway | 0.003 | 29 | 4 |
| Epithelial cell signalling in Helicobacter pylori infection | 0.004 | 27 | 4 |
| cGMP-PKG signalling pathway | 0.004 | 50 | 4 |
| mRNA surveillance pathway | 0.004 | 34 | 4 |
| Progesterone-mediated oocyte maturation | 0.004 | 33 | 4 |
| Hepatocellular carcinoma | 0.005 | 50 | 4 |
| EGFR tyrosine kinase inhibitor resistance | 0.006 | 27 | 4 |
| Inositol phosphate metabolism | 0.006 | 26 | 4 |
| Endocytosis | 0.006 | 79 | 4 |
| Cushing syndrome | 0.007 | 49 | 4 |
| Gastric acid secretion | 0.008 | 26 | 4 |
| Vibrio cholerae infection | 0.008 | 21 | 4 |
| Long-term depression | 0.008 | 22 | 4 |
| Bacterial invasion of epithelial cells | 0.008 | 26 | 4 |
| Dopaminergic synapse | 0.008 | 41 | 4 |
| Fc gamma R-mediated phagocytosis | 0.009 | 31 | 4 |
| Alanine, aspartate and glutamate metabolism | 0.009 | 15 | 4 |

^1^adj *p*-value: adjusted *p*-value using Benjamini-Hochberg false discovery rate correction.
